# Summary statistics-based association test for identifying the pleiotropic effects with set of genetic variants

**DOI:** 10.1093/bioinformatics/btad182

**Published:** 2023-04-07

**Authors:** Deliang Bu, Xiao Wang, Qizhai Li

**Affiliations:** School of Statistics, Capital University of Economics and Business, Beijing, China; School of Mathematics and Statistics, Qingdao University, Qingdao, China; LSC, NCMIS, Academy of Mathematics and Systems Science, Chinese Academy of Sciences, Beijing, China; School of Mathematical Sciences, University of Chinese Academy of Sciences, Beijing, China

## Abstract

**Motivation:**

Traditional genome-wide association study focuses on testing one-to-one relationship between genetic variants and complex human diseases or traits. While its success in the past decade, this one-to-one paradigm lacks efficiency because it does not utilize the information of intrinsic genetic structure and pleiotropic effects. Due to privacy reasons, only summary statistics of current genome-wide association study data are publicly available. Existing summary statistics-based association tests do not consider covariates for regression model, while adjusting for covariates including population stratification factors is a routine issue.

**Results:**

In this work, we first derive the correlation coefficients between summary Wald statistics obtained from linear regression model with covariates. Then, a new test is proposed by integrating three-level information including the intrinsic genetic structure, pleiotropy, and the potential information combinations. Extensive simulations demonstrate that the proposed test outperforms three other existing methods under most of the considered scenarios. Real data analysis of polyunsaturated fatty acids further shows that the proposed test can identify more genes than the compared existing methods.

**Availability and implementation:**

Code is available at https://github.com/bschilder/ThreeWayTest.

## 1 Introduction

Genome-wide association study (GWAS) has become a powerful tool for analyzing the association between genetic variants and complex human diseases and traits, such as hypertension (Franceschini et al. 2013), type 2 diabetes [DIAbetes Genetics Replication And Meta-analysis (DIAGRAM) Consortium 2014], and Alzheimer’s disease (Knutson and Pan 2021). The identification of these genetic variants helps reveal the biological insights of complex human diseases and traits. After the past decade’s development, several softwares, such as PLINK (Purcell et al. 2007), snptest (Marchini et al. 2007), MERLIN (Abecasis et al. 2002), AssocTest (Wang et al. 2020), and others are developed, and numerous public data (Leslie et al. 2014) are deposited. With these tools, scientists have been able to efficiently analyze data across different research labs and identify new disease-associated single nucleotide polymorphisms (SNPs).

Traditionally, GWAS tests the “one-to-one” association between phenotypes and SNPs. That is, it performs univariate regression analysis on one trait and one SNP while adapting the nominal α criterion level for extensive multiple testing, typically $\alpha =5\times 10^{-8}$. This univariate method may be inefficient for three reasons. First, phenotypically, several traits are often tied together to represent complex diseases. For example, a hypertension study often measures systolic blood pressure, diastolic blood pressure, and hypertension status (The International Consortium for Blood Pressure Genome-Wide Association Studies 2011). Recent studies also show the existence of pleiotropic effects which means that some SNPs are associated with multiple phenotypes simultaneously (Solovieff et al. 2013, Watanabe et al. 2019). For example, SNP rs11209026 was reported to be associated with four complex human diseases including Crohn’s disease (Duerr et al. 2006), ankylosing spondylitis [The Australo-Anglo-American Spondyloarthritis Consortium and the Wellcome Trust Case Control Consortium 2 2011], ulcerative colitis (Silverberg et al. 2009), and psoriasis (Genetic Analysis of Psoriasis Consortium and the Wellcome Trust Case Control Consortium2 2010). Second, genotypically, multiple variants may contribute to complex disease simultaneously, and each one has a low marginal effect that cannot be detected by single variant analysis (Madsen and Browning 2009). Third, statistically, group analysis of SNPs and phenotypes can, to some extent, increase statistical power. Consequently, many tests have been developed and they have proved to be more powerful than the traditional univariate test under some situations. These methods can be broadly classified into “one-to-multiple” methods testing the association between one phenotype and multiple variants, such as SKAT (Wu et al. 2011), C-alpha test (Neale et al. 2011), ACAT (Liu et al. 2019), and sum-based test (Han and Pan 2010), and “multiple-to-one” methods testing the association between multiple traits and a single variant, which contain principal component-based methods (Aschard et al. 2014, Zhang et al. 2017, Liu and Lin 2019), phenotype-correlation weighted methods (van der Sluis et al. 2013, Zhu et al. 2015), and others (Kim et al. 2015, Liu and Lin 2018, Ray and Chatterjee 2020, Liu et al. 2022, Wu et al. 2022a).

It should be noted that access to current GWAS individual data is restricted, owing to identification and privacy issues. Thus, only GWAS summary statistics data are available, and these data only contain Wald test statistics in linear regression models (Leslie et al. 2014). Thus, in this article, we focus on the development of a “multiple-to-multiple” test to identify the association between multiple traits and multiple variants based on GWAS summary statistics. To accomplish this, it is necessary to estimate the correlation matrix between summary statistics, which can be calculated by phenotype-correlation matrix and variant correlation matrix. In the absence of individual data, the sample correlation estimation method cannot be directly applied, complicating the estimation process compared to individual data-based tests. It is also necessary to consider the diverse structure of the data. Typically, if we want to test whether *m* SNPs are associated with *q* phenotypes, we need to combine the information of *qm* univariate Wald test statistics. The signals contained in these Wald test statistics may follow different structures under different situations. Also, a uniformly powerful test does not exist for a multidimensional composite alternative hypothesis (Liu and Lin 2019). Thus, we need to build a robust test statistic that can maintain a reasonable power in various situations. To solve these tasks, several different “multiple-to-multiple” methods based on summary statistics have already been proposed in the literature. For example, metaCCA uses canonical correlation analysis to test for association (Cichonska et al. 2016). MGAS (Van der Sluis et al. 2015) uses TATES (van der Sluis et al. 2013) method to combine the *P*-values of Wald test statistics. Guo and Wu (2019) proposed a variance component test, which can be seen as a combination of χ^2^ test and traditional sum test.

In this article, we propose a new test statistic that can fully utilize the diverse structures between SNPs and phenotypes. Guo and Wu (2019) provided the correlation matrix estimation procedure under the simplest situation of GWAS summary statistics via linear regression model, but without covariates. However, adjusting for covariates in a GWAS is both routine and necessary since the population stratification confounder factors may lead to many false-positive findings. Therefore, we obtain the correlation matrix estimation procedures of GWAS summary Wald statistics for linear regression model with covariates. Additionally, we prove that these *qm* univariate Wald statistics follow the multivariate normal distribution asymptotically, a property that has been naturally assumed without theoretical justification in the literature. Then by integrating three levels of information, including the intrinsic genetic structure, pleiotropy, and the potential information combinations, a test is proposed via Cauchy combination strategy. Extensive simulation results demonstrate that the proposed test can control type I error rates properly and retain reasonable power under most of the considered scenarios. We also apply the proposed test to polyunsaturated fatty acids data, and the results show that our method can identify more genes than the compared existing tests.

## 2 Methodology

### 2.1 Notations

Suppose that *q* phenotypes and *m* SNPs are included in a GWAS. For the *j*th phenotype and *k*th SNP, the summary statistics available publicly are frequently Wald test statistics $Z_{jk}$, j=1,2,…,q, k=1,2,….m. Denote M=qm, and we have *M* summary statistics in total which can be written in matrix form as


$$Z=\left(\begin{array}{cccc} Z_{11} & Z_{12} & \cdots & Z_{1m}\\ Z_{21} & Z_{22} & \cdots & Z_{2m}\\ \vdots & \vdots & \ddots & \vdots \\ Z_{q1} & Z_{q2} & \cdots & Z_{qm} \end{array}\right).$$

The null hypothesis, denoted by $H_{0}$, is that these *m* SNPs are not associated with *q* phenotypes, and the alternative hypothesis is that at least one SNP is associated with at least one phenotype. Let vec(Z) be the vectorized form of matrix Z stacked by column. Under $H_{0}$, from Supplementary Part 1 in the online supplementary material, we show that vec(Z) follows a multivariate normal distribution with zero mean vector and its covariance matrix will be given later. Our aim is to utilize Z to construct a powerful and robust test to check whether $H_{0}$ holds or not.

### 2.2 Correlation coefficient estimation procedure with covariates

Estimating the covariance matrix of vec(Z) can be directly transformed into estimating the pairwise correlation coefficient between two elements in vec(Z) since each $Z_{jk}$ has the asymptotic variance of one under $H_{0}$. To simplify the notation, we consider two Wald test statistics $Z_{jk}$ and $Z_{j^{\prime }k^{\prime }}$ separately obtained from different quantitative traits $Y_{j}$ and $Y_{j^{\prime }}$ and different SNPs $G_{k}$ and $G_{k^{\prime }}$ while adjusting for covariates *C*, where $j,j^{\prime }=1,2,\ldots ,q$ and $k,k^{\prime }=1,2,\ldots ,m$. From Supplementary Part 2 in the online supplementary material, we can conclude that where $\rho_{jj^{\prime }}$ is the correlation coefficient between $Y_{j}$ and $Y_{j^{\prime }}$ and $\theta_{kk^{\prime }}$ is partial correlation coefficient between $G_{k}$ and $G_{k^{\prime }}$ given covariates *C*. So, estimating $\text{corr}(Z_{jk},Z_{j^{\prime }k^{\prime }})$ is transformed to estimate $\rho_{jj^{\prime }}$ and $\theta_{kk^{\prime }}$.


$$\text{corr}(Z_{jk},Z_{j^{\prime }k^{\prime }})=\rho_{jj^{\prime }}\theta_{kk^{\prime }},$$

The estimation procedure of $\rho_{jj^{\prime }}$ has already been well studied in the literature (Zhu et al. 2015, Liu and Lin 2018) and can be described as follows. Suppose, we have *L* independent null SNPs at hand, and *L* pairs of Wald tests from phenotypes *j* and $j^{\prime }$ as $\left(z_{jl},z_{j^{\prime }l}\right)$ for l=1,2,…,L, we can estimate $\rho_{jj^{\prime }}$ using where ${\bar{z}}_{j}=\frac{1}{L}\sum_{l=1}^{L}z_{jl}$ and ${\bar{z}}_{j^{\prime }}=\frac{1}{L}\sum_{l=1}^{L}z_{j^{\prime }l}$.


$${\hat{\rho }}_{jj^{\prime }}=\frac{\sum_{l=1}^{L}\left(z_{jl}-{\bar{z}}_{j}\right)(z_{j^{\prime }l}-{\bar{z}}_{j^{\prime }})}{\sqrt{\sum_{l=1}^{L}{\left(z_{jl}-{\bar{z}}_{j}\right)}^{2}}\sqrt{\sum_{l=1}^{L}{\left(z_{j^{\prime }l}-{\bar{z}}_{j^{\prime }}\right)}^{2}}},$$

To estimate the partial correlation coefficient of $\theta_{kk^{\prime }}$, three cases are considered. In the first case, all covariates *C* are environment variables. Because of the independence between environment variables and genetic variants, estimating $\theta_{kk^{\prime }}$ is reduced to estimating the Pearson correlation coefficient between $G_{k}$ and $G_{k^{\prime }}$. Denote it by $r_{kk^{\prime }}$. In this case, the result is the same as that for the situation with no covariates. With only summary statistics at hand, $r_{kk^{\prime }}$ cannot be calculated directly. However, $r_{kk^{\prime }}$ does not depend on individual sample (Bulik-Sullivan et al. 2015, Cichonska et al. 2016). When the target population information is available, which is usually the case in practice, $r_{kk^{\prime }}$ can be estimated by reference dataset from target population. For example, one can use 1000 Genomes Projects (1000 Genomes Project Consortium 2015) as reference panel, which is commonly used in genetic association analysis (Bulik-Sullivan et al. 2015, Van der Sluis et al. 2015, Cichonska et al. 2016). Suppose that there are *N* subjects with genotypes $g_{ik}^{*}$ and $g_{ik^{\prime }}^{*}$ for SNPs $G_{k}$ and $G_{k}^{\prime }$, respectively, i=1,2,…,N, in a reference dataset. Then, the estimate of $r_{kk^{\prime }}$ is where ${\bar{g}}_{k}^{*}=\frac{1}{N}\sum_{i=1}^{N}g_{ik}^{*}$ and ${\bar{g}}_{k^{\prime }}^{*}=\frac{1}{N}\sum_{i=1}^{N}g_{ik^{\prime }}^{*}$.


$${\hat{r}}_{kk^{\prime }}=\frac{\sum_{i=1}^{N}\left(g_{ik}^{*}-{\bar{g}}_{k}^{*}\right)(g_{ik^{\prime }}^{*}-{\bar{g}}_{k^{\prime }}^{*})}{\sqrt{\sum_{i=1}^{N}{\left(g_{ik}^{*}-{\bar{g}}_{k}^{*}\right)}^{2}}\sqrt{\sum_{i=1}^{N}{\left(g_{ik^{\prime }}^{*}-{\bar{g}}_{k^{\prime }}^{*}\right)}^{2}}},$$

In the second case, all covariates *C* are population stratification confounders. Similar to Case 1, we can use a reference dataset from target population, such as 1000 Genomes Projects to extract *s* (e.g. s=2 or 5) principal components or principal coordinates (Price et al. 2006, Li and Yu 2008) using R functions “MDS” or “PCoC” in R package “AssocTest” as *C*. We then regress $G_{k}$ and $G_{k^{\prime }}$ on *C* separately to get their residuals, and calculate the Pearson correlation coefficient between these residuals as the estimate of $\theta_{kk^{\prime }}$. In detail, suppose that there are *N* subjects with genotypes $g_{ik}^{*}$ and $g_{ik^{\prime }}^{*}$ for SNPs $G_{k}$ and $G_{k^{\prime }}$, respectively, i=1,2,…,N, in a reference dataset. Let $g_{k}^{*}={\left(g_{1k}^{*},g_{2k}^{*},\ldots ,g_{Nk}^{*}\right)}^{⊤}$, $g_{k^{\prime }}^{*}={\left(g_{1k^{\prime }}^{*},g_{2k^{\prime }}^{*},\ldots ,g_{Nk^{\prime }}^{*}\right)}^{⊤}$, C be N×(s+1) design matrix formulated by the chosen principal components or principal coordinates, where the superscript ${}^{⊤}$ denotes the transpose of a matrix or a vector, and the first column of C is all equal to 1, which represents the intercept. We then calculate where $I_{n}$ is the *n*-dimensional identity matrix. Denote $h_{k}^{*}={\left(h_{1k}^{*},h_{2k}^{*},\ldots ,h_{Nk}^{*}\right)}^{⊤}$ and $h_{k^{\prime }}^{*}={\left(h_{1k^{\prime }}^{*},h_{2k^{\prime }}^{*},\ldots ,h_{Nk^{\prime }}^{*}\right)}^{⊤}$. The estimate of $\theta_{kk^{\prime }}$ is where ${\bar{h}}_{k}^{*}=\frac{1}{N}\sum_{i=1}^{N}h_{ik}^{*}$ and ${\bar{h}}_{k^{\prime }}^{*}=\frac{1}{N}\sum_{i=1}^{N}h_{ik^{\prime }}^{*}$.


$$h_{k}^{*}=(I_{n}-C{\left(C^{⊤}C\right)}^{-1}C^{⊤})g_{k}^{*},\;\;h_{k^{\prime }}^{*}=(I_{n}-C{\left(C^{⊤}C\right)}^{-1}C^{⊤})g_{k^{\prime }}^{*},$$

$${\hat{\theta }}_{kk^{\prime }}=\frac{\sum_{i=1}^{N}\left(h_{ik}^{*}-{\bar{h}}_{k}^{*}\right)(h_{ik^{\prime }}^{*}-{\bar{h}}_{k^{\prime }}^{*})}{\sqrt{\sum_{i=1}^{N}{\left(h_{ik}^{*}-{\bar{h}}_{k}^{*}\right)}^{2}}\sqrt{\sum_{i=1}^{N}{\left(h_{ik^{\prime }}^{*}-{\bar{h}}_{k^{\prime }}^{*}\right)}^{2}}}.$$

In the third case, the covariates consist of both the environmental variables and the population stratification confounders. Since the environmental variables are independent of the genetic variants, we can ignore the environmental variables and the estimation procedure directly equals to the second case for estimating $\theta_{kk^{\prime }}$.

### 2.3 Test statistic

Denote


$$\Delta_{\rho }=\left(\begin{array}{cccc} 1 & {\hat{\rho }}_{12} & \cdots & {\hat{\rho }}_{1q}\\ {\hat{\rho }}_{12} & 1 & \cdots & {\hat{\rho }}_{2q}\\ \vdots & \vdots & \ddots & \vdots \\ {\hat{\rho }}_{1q} & {\hat{\rho }}_{2q} & \cdots & 1 \end{array}\right),\;\;\Delta_{\theta }=\left(\begin{array}{cccc} 1 & {\hat{\theta }}_{12} & \cdots & {\hat{\theta }}_{1m}\\ {\hat{\theta }}_{12} & 1 & \cdots & {\hat{\theta }}_{2m}\\ \vdots & \vdots & \ddots & \vdots \\ {\hat{\theta }}_{1m} & {\hat{\theta }}_{2m} & \cdots & 1 \end{array}\right).$$

Based on Supplementary Parts 1 and 2 in the online supplementary material, under $H_{0}$, ${\left(\Delta_{\theta }⊗\Delta_{\rho }\right)}^{-\frac{1}{2}}\text{vec}(Z)$ asymptotically follows $N(0_{M},I_{M}),$$0_{M}$ is a M−dimensional column vector with all zero units, and ⊗ denotes the Kronecker product. Now, we will build a test considering three-way information including intrinsic genetic structure, pleiotropy, and potential information combinations.

First consider intrinsic genetic structure. Because the Wald test statistics capture the genetic effects of all SNPs in linkage disequilibrium (Chun et al. 2020), one deleterious variant would lead to multiple non-zero Wald test statistics in a particular region. So, integrating the *j*th row of Z into one statistic might lead to a high power. Denote the *j*th row of matrix Z as a column vector $Z_{\left\{j\right\}}$, i.e. $Z_{\left\{j\right\}}={\left(Z_{j1},Z_{j2},\ldots ,Z_{jm}\right)}^{⊤}$, j=1,2,…,q. A statistic for grasping the association between the *j*th phenotype and *m* SNPs is $Z_{\left\{j\right\}}^{⊤}\Delta_{\theta }^{-1}Z_{\left\{j\right\}}$, j=1,2,…,q. Under $H_{0}$, $Z_{\left\{j\right\}}^{⊤}\Delta_{\theta }^{-1}Z_{\left\{j\right\}}$ asymptotically follow $\chi_{m}^{2},$ a χ^2^ distribution with *m* degrees of freedom. Denote the *P*-value of *j*th phenotype as $p_{j},j=1,2,\ldots q$. To combine these effects over *q* phenotypes, we use the Cauchy combination strategy (Liu and Xie 2020, Long et al. 2022). It can be showed that under certain conditions the sum of some class of dependent Cauchy variables approximately follows Cauchy distribution in the tail. Since $p_{j},j=1,2,\ldots q$ follows uniform distribution between 0 and 1 under the null, each $p_{j}$ can be transformed into following a standard Cauchy distribution by $\text{tan}\;\{(0.5-p_{j})\pi \}$. Based on this, Liu and Xie (2020) and Long et al. (2022) constructed Cauchy Combination Test (CCT) as where $\omega_{j}$ is non-negative weight and $\sum_{j=1}^{q}\omega_{j}=1$. They then proved that under the null hypothesis, where Cauchy(0,1) stands for a standard Cauchy distributed variable. This result demonstrates that the tail of CCT asymptotically follows standard Cauchy distribution under arbitrary correlation structure. Then, the *P*-value of the CCT can be easily approximated by


$$\text{CCT}=\sum_{j=1}^{q}\omega_{j}\;\text{tan}\;\{(0.5-p_{j})\pi \}.$$

$$\underset{t\to \infty }{\lim}\frac{P\{\text{CCT}>t\}}{P\{\text{Cauchy}(0,1)>t\}}=1,$$

P-value≈0.5−{arctan(CCT)}/π.

The power of CCT is basically the same as minimal *P*-value test in most situation without using any resampling procedure (Liu and Xie 2020, Long et al. 2022). This theoretical result suggests that the Cauchy distribution can be used to combine the *P*-values under arbitrary correlation structure. We can directly apply CCT to the *P*-values $p_{1},p_{2},\ldots ,p_{q}$. The final test statistic of $T_{1}$ is which follows standard Cauchy distribution under the null. Its *P*-value can be easily calculated by


$$T_{1}=\sum_{j=1}^{q}\frac{1}{q}\;\text{tan}\;\{(0.5-p_{j})\pi \},$$

$$P_{1}=0.5-\{\text{arctan}(T_{1})\}/\pi .$$

Next, we consider the pleiotropic effect. Denote the *k*th column of Z as $Z_{\left[k\right]}={\left(Z_{1k},Z_{2k},\ldots ,Z_{qk}\right)}^{⊤}$. Then $Z_{\left[k\right]}^{⊤}\Delta_{\rho }^{-1}Z_{\left[k\right]}$ asymptotically follows $\chi_{q}^{2}$ under $H_{0}$, the *P*-value of which is denoted by $p_{\left[k\right]}$, k=1,2,…,m Then, the second-way statistic is $T_{2}=\sum_{k=1}^{m}\frac{1}{m}\;\text{tan}((0.5-p_{\left[k\right]})\pi )$, the statistical significance of which has a closed form as $P_{2}=0.5-\text{arctan}(t_{2})/\pi ,$ where $t_{2}$ is the observed value of $T_{2}$.

Last, we consider the potential information combinations. Although the first and second ways for combining signals are straight forward and deal with two typical situations in GWAS. However, not all signals follow the aforementioned two situations dealing by the first and second way. Sometimes the signals can be irregular or a combination of the first and second ways. In this situation, the first and second ways for combining the signals will lose power. Thus, we incorporate the third way of combining the signals, which can be seen as a complement to make our statistics more robust. The idea is to select the subset of $\text{vec}(Z)=(Z_{11},Z_{21},\ldots ,Z_{qm})$ based on its absolute value to construct a statistic. We first rank $\left|Z_{11}|,|Z_{21}|,\ldots ,|Z_{qm}\right|$ in a descending order, and denote them as $Z_{\left(1\right)},Z_{\left(2\right)},\ldots ,Z_{\left(M\right)}$, where $Z_{\left(1\right)}$ and $Z_{\left(M\right)}$ represent the maximum and minimum values, respectively. Define a constant η∈(0,1]. Then, a statistic is where $Z_{\eta }$ is the subvector of vec(Z) indexed by $Z_{\left(1\right)},Z_{\left(2\right)},\ldots ,Z_{\left(\left\lceil \eta M\right\rceil \right)}$, in which ⌈x⌉ indicates the minimum integer larger than *x* with x>0, and $\Delta_{\eta }$ is the submatrix of $\Delta_{\theta }⊗\Delta_{\rho }$ corresponding to $Z_{\eta }$. Since the optimal η is not known beforehand, we choose η from a set Ω={0.1,0.2,…,0.9,1} and combine ${\tilde{T}}_{\eta }$s using Cauchy combination strategy. Denote the *P*-value of ${\tilde{T}}_{\eta }$ by ${\tilde{p}}_{\eta },\eta \in \Omega$, with calculation given in next section. Then, the third-way statistic is $T_{3}=\sum_{\eta \in \Omega }\frac{1}{\#\Omega }\;\text{tan}((0.5-{\tilde{p}}_{\eta })\pi )$. The statistical significance of which has a closed form as $P_{3}=0.5-\text{arctan}(t_{3})/\pi ,$ here, #Ω denotes the number of elements in Ω and $t_{3}$ is observed value of $T_{3}$.


(1) $${\tilde{T}}_{\eta }=Z_{\eta }^{⊤}\Delta_{\eta }^{-1}Z_{\eta },$$

With the above three-way statistics, the final test, which is named as Three-Way Test (TWT) can be calculated as the *P*-value of which is $0.5-\{\text{arctan}(t_{0})\}/\pi ,$ where $t_{0}$ is the observed value of TWT.


$$\text{TWT}=\sum_{i=1}^{3}\frac{1}{3}\;\text{tan}((0.5-P_{i})\pi ),$$

### 2.4 *P*-value calculation of $P_{3}$

The proposed test is a combination of three *P*-values, $P_{1},P_{2},$ and $P_{3}$, where the calculations of $P_{1}$ and $P_{2}$ are rather straightforward. Now, we describe a fast parameter bootstrap procedure to get $P_{3}.$

*A parameter bootstrap procedure*:

*Step 1.* Estimate the correlation coefficient matrix of vec(Z) and denote it by $\Delta_{\theta }⊗\Delta_{\rho }$.

*Step 2.* Generate *B* independent and identical observations from $N(0_{M},\Delta_{\theta }⊗\Delta_{\rho })$, and arrange the *i*th observation with i=1,2,…,B in matrix form as


$$Z^{\left(i\right)}=\left(\begin{array}{cccc} Z_{11}^{\left(i\right)} & Z_{12}^{\left(i\right)} & \cdots & Z_{1m}^{\left(i\right)}\\ Z_{21}^{\left(i\right)} & Z_{22}^{\left(i\right)} & \cdots & Z_{2m}^{\left(i\right)}\\ \vdots & \vdots & \ddots & \vdots \\ Z_{q1}^{\left(i\right)} & Z_{q2}^{\left(i\right)} & \cdots & Z_{qm}^{\left(i\right)} \end{array}\right).$$

*Step 3.* For η∈Ω, calculate ${\tilde{T}}_{\eta }$ and denote it by ${\tilde{T}}_{\eta }^{\left(i\right)}$, i=1,2,…,B.

*Step 4.* Use the cumulants estimation proposed by Zhang (2005) and Li et al. (2014) to obtain the parameters of generalized χ^2^ distribution $a\chi_{d}^{2}+b$. Let ${\tilde{T}}_{\eta }^{\left(i\right)}$, η=0.1,0.2,…,0.9,i=1,2,…,B be random samples generated from generalized χ^2^ distribution $a\chi_{d}^{2}+b$. Define $K_{\eta }=\frac{1}{B}\sum_{i=1}^{B}{\tilde{T}}_{\eta }^{\left(i\right)}$, $L_{\eta }=\frac{1}{B}\sum_{i=1}^{B}{\left({\tilde{T}}_{\eta }^{\left(i\right)}-K_{\eta }\right)}^{2},$ and $M_{\eta }=\frac{1}{B}\sum_{i=1}^{B}{\left({\tilde{T}}_{\eta }^{\left(i\right)}-K_{\eta }\right)}^{3}$. Matching the first three cumulants, we have $a_{\eta }=M_{\eta }/4L_{\eta },b_{\eta }=K_{\eta }-2L_{\eta }^{2}/M_{\eta },$ and $d_{\eta }=8L_{\eta }^{3}/M_{\eta }^{2}$. Denote them as $\{a_{\eta },b_{\eta },d_{\eta }\},\eta =0.1,0.2,\ldots ,0.9$.

*Step 5.* Calculate the *P*-values ${\tilde{p}}_{\eta },\eta \in \Omega$ based on observed data, where ${\tilde{p}}_{1}$ can be calculated with traditional χ^2^ test. Other *P*-values can be generated by first calculating ${\tilde{T}}_{\eta },\eta =0.1,0.2,\ldots ,0.9$ as mentioned in Equation (1). Then by using the parameters $\{a_{\eta },b_{\eta },d_{\eta }\},\eta =0.1,0.2,\ldots ,0.9$ obtained in *Step 4.*, we can calculate the *P*-values by where η=0.1,0.2,…,0.9


$${\tilde{p}}_{\eta }=P(a_{\eta }\chi_{d_{\eta }}^{2}+b_{\eta }>{\tilde{T}}_{\eta }).$$

*Step 6.* $P_{3}$ is given by $P_{3}=0.5-\text{arctan}(t_{3})/\pi ,$ where $t_{3}=\sum_{\eta \in \Omega }\frac{1}{\#\Omega }\;\text{tan}((0.5-{\tilde{p}}_{\eta })\pi )$.

## 3 Simulation

We conduct simulation study to investigate the performances of the proposed TWT by comparing its type I error rates and powers with three other methods including metaCCA (Cichonska et al. 2016), MGAS (Van der Sluis et al. 2015), and MAT (Guo and Wu 2019).

### 3.1 Simulation settings

Generally speaking, we produce individual data and calculate Wald test statistics $Z_{jk},j=1,2,\ldots ,q,k=1,2,\ldots ,m$. The model for generating individual data can be described as follows: where $g_{ik}$ represents the genotype value, $\alpha_{j}$ is the intercept term, $\epsilon_{ij}$ is the error term, and $\beta_{jk}$ is the coefficient. Denote B as the matrix of regression coefficient with *q* rows and *m* columns, where elements of *j*th row and *k*th column are $\beta_{jk},j=1,2,\ldots ,q,k=1,2,\ldots ,m$. Without loss of generality, we assume there is 1D covariate $c_{i}$ and $\gamma_{j}$ is its regression coefficient. To mimic the reality, the genotype data $g_{ik}$ are directly taken from 1000 Genomes Project. Since the number of individual data are fixed in 1000 Genomes Project, the sample size is herein fixed to be n=2504. The other parameters that are changed under different scenarios include: *m*, $\Delta_{\theta }$, *q*, and $\Delta_{\rho }$. We will introduce the simulation settings one by one. For *m* and $\Delta_{\theta }$, the genotypes from four different genes directly annotated from the real data of polyunsaturated fatty acids are used. These genes are FADS2 with m=9, GNB1 with m=5, SGIP1 with m=50, and ACOT7 with m=6. For the phenotype dimension *q*, we consider two scenarios of q=6 and 10. When q=6, we set the correlation structure between traits $\Delta_{\rho }$ to be identical to the estimated correlation structure from real data. For the situation of q=10, we set the correlation matrix $\Delta_{\rho }$ to be the commonly used auto-regressive correlation matrix as $\Delta_{\rho }={\left(\rho^{\left|i-j\right|}\right)}_{q\times q}$ with ρ=0.1,0.5, and 0.8. For the regression model coefficient $\beta_{jk}$, under the null hypothesis, it is clear that all $\beta_{jk}$s equal 0, we denote it as “NONE.” Under the alternative hypothesis, we consider three non-zero signal allocations as follows: “SINGLE,” “ROW,” and “COLUMN,” where “SINGLE” means that only one elements in $\beta_{jk}$ is not zero, “ROW” means that the non-zero elements of $\beta_{jk}$ are spread over one particular row in B and “COLUMN” means that the non-zero elements of $\beta_{jk}$ are spread over one particular column. Note that the value of non-zero $\beta_{jk}$ is changed so that all methods have comparable power. Detailed scenario configurations and parameter combinations can be found in Supplementary Tables S1–S3 in Part 4 of online supplementary material. Supplementary Table S1 contains six scenarios: $S_{1-1}$ to $S_{2-3}$, all under the null hypothesis. Supplementary Tables S2 and S3 contain the scenario settings under the alternative hypothesis, in which Supplementary Table S2 contains scenarios $S_{1-1-1}$ to $S_{1-3-3}$ and Supplementary Table S3 contains scenario $S_{2-1-1}$ to $S_{2-3-3}$. The first two numbers of scenarios under alternative hypothesis correspond to the scenarios under null hypothesis. For example, $S_{1-1-1}$ has the same scenario configurations as $S_{1-1}$ except for the regression coefficient $\beta_{jk}$. Finally, we calculate the *M* Wald test statistics $Z_{jk}$ based on the generated individual data. To remove the effect of population stratification, we used 1000 Genomes Project data to extract top two principal coordinates with selected SNPs to calculate population stratification (Li and Yu 2008). Then used them as covariates in the regression model. We then applied the aforementioned four methods to combine these *M* Wald test statistics and calculate the empirical type I error rates and empirical powers based on $10^{6}$ replications. The significance level was chosen as $10^{-3}$ and $10^{-5}$.


$$y_{ij}=\alpha_{j}+\sum_{k=1}^{m}g_{ik}\beta_{jk}+c_{i}\gamma_{j}+\epsilon_{ij},i=1,2,\ldots ,n,j=1,2,\ldots ,q,$$

### 3.2 Simulation results

Table 1 presents the results of type I error rates of metaCCA, MGAS, MAT, and TWT under two significance levels of $10^{-3}$ and $10^{-5}$. From the table, we can see that all methods can control type I error rates properly. For example, under scenario $S_{1-1}$, the empirical type I error rates of metaCCA, MGAS, MAT, and TWT are $1.0\times 10^{-3}$, $1.2\times 10^{-3}$, $1.2\times 10^{-3}$, and $1.1\times 10^{-3}$, respectively.

**Table 1.** Empirical type I error rates of metaCCA, MGAS, MAT, and TWT under scenarios $S_{1-1}$ to $S_{2-3}$^a^.

Model	α	metaCCA	MGAS	MAT	TWT
$S_{1-1}$	$10^{-3}$	$1.0\times 10^{-3}$	$1.2\times 10^{-3}$	$1.2\times 10^{-3}$	$1.1\times 10^{-3}$
	$10^{-5}$	$1.3\times 10^{-5}$	$1.8\times 10^{-5}$	$1.0\times 10^{-5}$	$6.0\times 10^{-6}$
$S_{1-2}$	$10^{-3}$	$1.0\times 10^{-3}$	$1.2\times 10^{-3}$	$1.3\times 10^{-3}$	$1.1\times 10^{-3}$
	$10^{-5}$	$6.0\times 10^{-6}$	$7.0\times 10^{-6}$	$1.9\times 10^{-5}$	$1.1\times 10^{-5}$
$S_{1-3}$	$10^{-3}$	$1.0\times 10^{-3}$	$1.3\times 10^{-3}$	$1.1\times 10^{-3}$	$1.0\times 10^{-3}$
	$10^{-5}$	$9.0\times 10^{-6}$	$1.6\times 10^{-5}$	$2.1\times 10^{-5}$	$1.2\times 10^{-5}$
$S_{2-1}$	$10^{-3}$	$1.1\times 10^{-3}$	$1.3\times 10^{-3}$	$1.1\times 10^{-3}$	$1.1\times 10^{-3}$
	$10^{-5}$	$1.0\times 10^{-5}$	$1.0\times 10^{-5}$	$7.0\times 10^{-6}$	$8.0\times 10^{-6}$
$S_{2-2}$	$10^{-3}$	$1.0\times 10^{-3}$	$1.4\times 10^{-3}$	$1.2\times 10^{-3}$	$1.2\times 10^{-3}$
	$10^{-5}$	$1.4\times 10^{-5}$	$1.3\times 10^{-5}$	$1.3\times 10^{-5}$	$1.3\times 10^{-5}$
$S_{2-3}$	$10^{-3}$	$1.1\times 10^{-3}$	$1.3\times 10^{-3}$	$1.1\times 10^{-3}$	$1.2\times 10^{-3}$
	$10^{-5}$	$9.0\times 10^{-6}$	$2.1\times 10^{-5}$	$8.0\times 10^{-6}$	$1.1\times 10^{-5}$

a The significance level α is set to $10^{-3}$ and $10^{-5}$.

**Figure 1:**
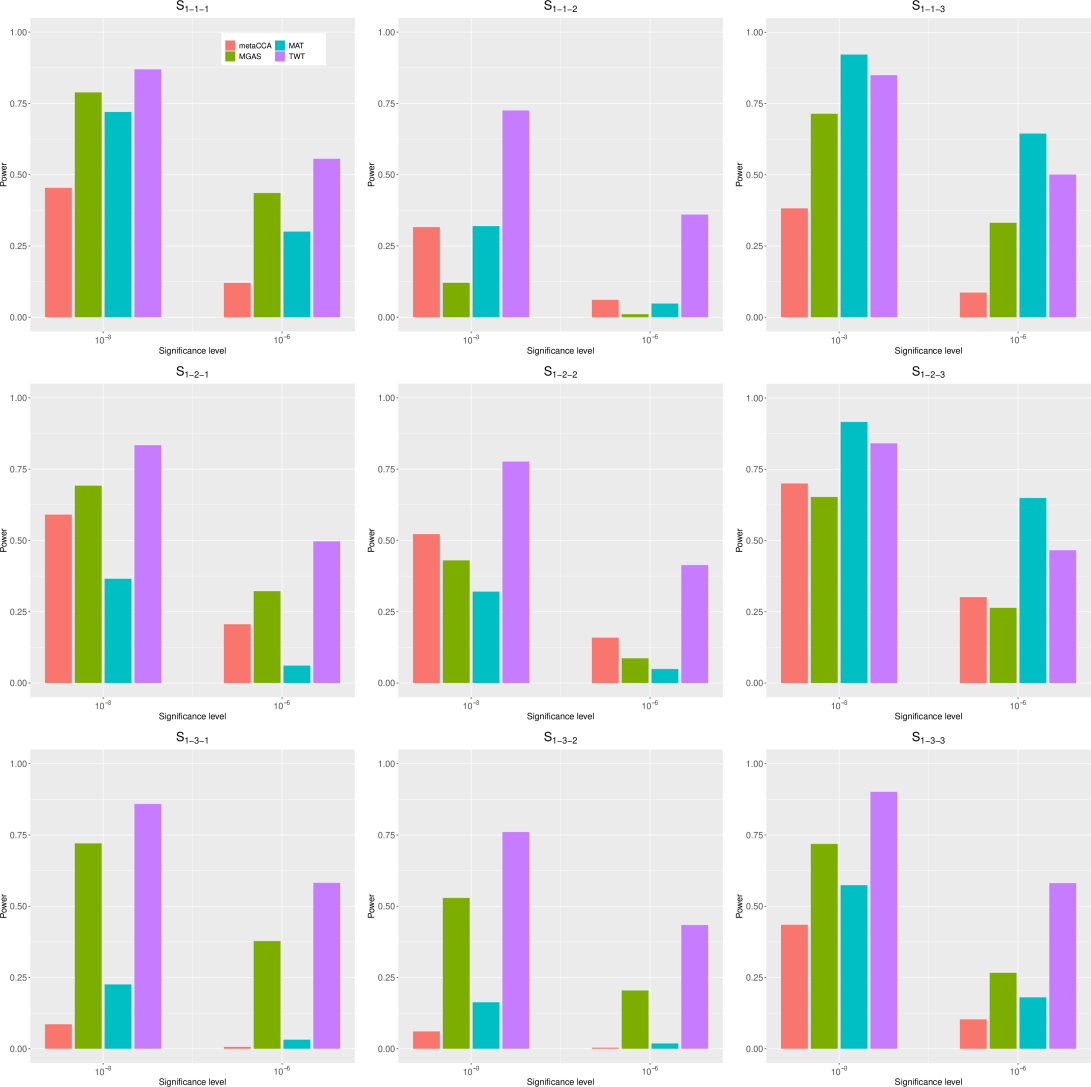
Empirical powers of metaCCA(red), MGAS (green), MAT (blue), and TWT (purple) under scenarios $S_{1-1-1}$ to $S_{1-3-3}$

**Figure 2:**
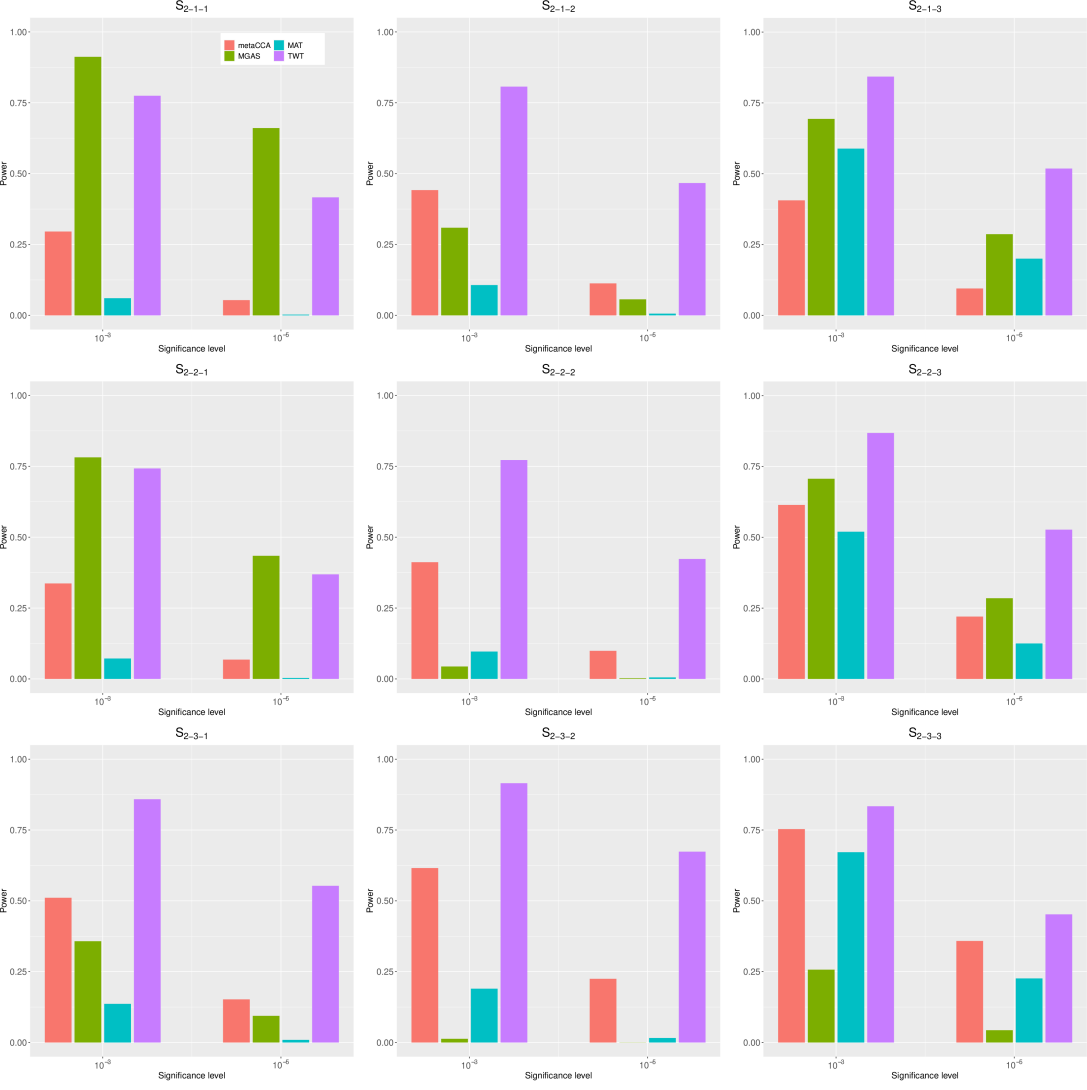
Empirical powers of metaCCA(red), MGAS (green), MAT (blue), and TWT (purple) under scenarios $S_{2-1-1}$ to $S_{2-3-3}$

Figure 1 shows the empirical powers of metaCCA, MGAS, MAT, and TWT under scenarios $S_{1-1-1}$ to $S_{1-3-3}$ and Fig. 2 shows the empirical powers of the aforementioned four methods under scenarios $S_{2-1-1}$ to $S_{2-3-3}$.

From the figures, we can conclude that our method either produces the most powerful results or has power similar to that of the most powerful method while all other three methods lost power drastically under some scenarios. TWT generates powerful results when the pleiotropic effect exist, i.e. the non-zero elements in $\beta_{jk}$ lies in one particular column of B. For example, in scenario $S_{2-1-2}$, under significance level of $10^{-3}$, the empirical power of TWT is 0.81, while the empirical powers of metaCCA, MGAS, and MAT are 0.44, 0.31, and 0.10, respectively. The power increment of TWT is at least 0.37 compared with other three methods. As single elements in B is non-zero, under scenario $S_{2-1-1}$, MGAS can also generate powerful results. Under significance level of $10^{-3}$, the power of MGAS is 0.91 and the power of TWT is 0.77 whereas the power of metaCCA and MAT is only 0.30 and 0.06, respectively, much lower than MGAS and TWT. This result is reasonable since MGAS can be seen as a modified minimum *P*-value methods, which generates powerful results when there is only one non-zero element. However, the power of MGAS decreases quickly with the increment of the correlation coefficient between phenotypes. Under scenario $S_{2-2-1}$, with ρ=0.5, the empirical powers of MGAS and TWT are nearly equal. When ρ is set to be 0.8, under scenario $S_{2-3-1}$, TWT is much more powerful than MGAS with the empirical power of 0.86 under significance level of $10^{-3}$, whereas that of MGAS is only 0.36. When multiple variants contribute to one particular phenotype, i.e. the non-zero elements lie in one particular row of B, MAT and TWT produce most powerful results with similar power. For example, under scenario $S_{1-1-3}$, with significance level of $10^{-3}$, the power of TWT is 0.85, the power of MAT is 0.92 whereas the powers of metaCCA and MGAS are 0.38 and 0.71. Overall, the proposed TWT has a reasonable power over the considered scenarios and is much more robust than other three methods.

## 4 Real data analysis

We analyzed the summary statistics data from the polyunsaturated fatty acids study conducted by Tanaka et al. (2009). Polyunsaturated fatty acids refer to the class of fatty acids with multiple desaturations in the aliphatic tail. Existing studies have shown that the fatty acid and plasma levels are associated with reduced risk of cardiovascular disease (Hu et al. 1999), depression (Tanskanen et al. 2001), and diabetes (Hodge et al. 2007). The polyunsaturated fatty acids study consists of six phenotypes including linoleic acid, arachidonic acid, eicosadienoic acid, alpha-linolenic acid, eicosapentanoic acid, and docosahexanoic acid. Summary Wald test statistics of these six phenotypes based on different SNPs were reported in the public access data on the website at https://grasp.nhlbi.nih.gov/.

The original data were collected based on SNPs and 495 343 SNPs were included. To perform the multiple-to-multiple test, these SNPs were located in different genes with ±5kb regions. When a SNP was located in overlapping regions of multiple genes, the SNP was assigned to all involved genes. We then removed SNPs for which the count allele and alternative allele were not identical between 1000 Genomes Project and Polyunsaturated Fatty Acids summary statistics data and genes with extremely large and small numbers of SNPs. After data pre-processing, 12 984 genes were included for further analysis.

Population stratification was previously adjusted for in the calculation of original summary statistics, Thus, the calculation of correlation matrix between Wald test statistics belongs to case two as described in our correlation estimation section. Following the estimation method mentioned in correlation estimation section, we use 1000 Genomes Project data to extract top two principal coordinates with selected SNPs used to calculate population stratification (Li and Yu 2008) and used them to calculate the genotype correlation matrix $\Delta_{\theta }$ using the residuals. Phenotype-correlation matrix $\Delta_{\rho }$ was calculated using the summary Wald test statistics via independent SNP with *P*-values >.05 (Kim et al. 2015, Kwak and Pan 2017).

**Figure 3:**
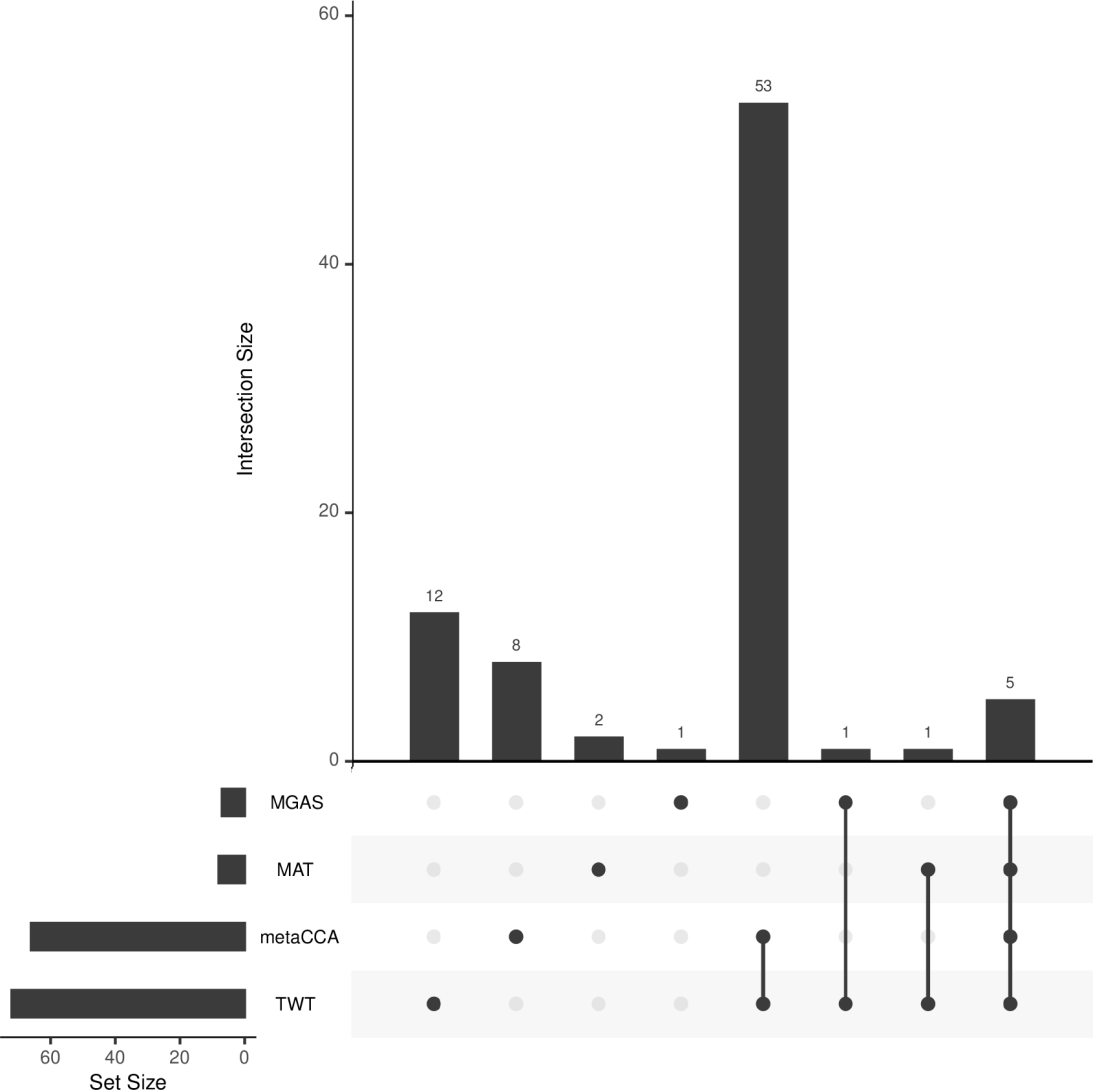
The upset plot of significant genes detected by metaCCA, MGAS, MAT, and TWT

Bonferroni correction was used to evaluate the significance. Since 12 984 genes were included, *P*-values <$3.85\times 10^{-6}(\approx 0.05/12984)$ could be reported to be associated. metaCCA, MGAS, MAT, and TWT identified 66, 7, 8, and 72 genes, respectively. It can be seen that the proposed TWT identifies the most significant genes, and metaCCA identifies 66 genes, which is close to TWT. The other two methods miss many findings in our analysis. The upset plot of the number of significant genes identified by the proposed method and other three methods can be found in Fig. 3.

From the figure, we can see that 12 genes are only detected significantly by our proposed method TWT. *P*-values of these 12 genes can be found in Table 2, and all significant genes detected by TWT can be found in Supplementary Part 6 in the online supplementary material. Note that due to the numerical precision limits of the built in functions ‘tan’ and ‘atan’ in R, our new method cannot generate *P*-values smaller than $10^{-14}$, so these *P*-values are replaced as $<10^{-14}$ in the tables. This limitation does not affect the practical use of our new method since we are mostly concerned with whether SNPs or genes reach genome-wide association level, not the exact value extremely small *P*-values.

**Table 2.** *P*-value of genes that are only detect to have the associations by TWT under the significant level of $3.85\times 10^{-6}(\approx 0.05/12\;984)$.

Gene	metaCCA	MGAS	MAT	TWT
LINC01226	$5.13\times 10^{-5}$	$9.00\times 10^{-1}$	$3.08\times 10^{-1}$	$6.60\times 10^{-7}$
GNG12	$4.13\times 10^{-4}$	$2.88\times 10^{-1}$	$8.82\times 10^{-1}$	$6.47\times 10^{-10}$
CAPZA1	$3.08\times 10^{-4}$	$2.82\times 10^{-1}$	$6.45\times 10^{-1}$	$1.96\times 10^{-6}$
SRP72	$1.91\times 10^{-5}$	$4.14\times 10^{-2}$	$3.09\times 10^{-2}$	$9.25\times 10^{-8}$
AQPEP	$3.93\times 10^{-6}$	$6.34\times 10^{-1}$	$3.50\times 10^{-1}$	$3.61\times 10^{-9}$
CPEB4	$8.56\times 10^{-6}$	$2.78\times 10^{-1}$	$1.71\times 10^{-1}$	$1.24\times 10^{-6}$
EGFR	$3.68\times 10^{-5}$	$7.92\times 10^{-1}$	$6.33\times 10^{-1}$	$3.17\times 10^{-11}$
KAT6A	$1.45\times 10^{-3}$	$4.42\times 10^{-1}$	$2.65\times 10^{-1}$	$5.41\times 10^{-7}$
DPYS	$1.85\times 10^{-3}$	$7.82\times 10^{-3}$	$5.43\times 10^{-3}$	$1.32\times 10^{-6}$
EML5	$3.37\times 10^{-5}$	$9.03\times 10^{-2}$	$7.83\times 10^{-4}$	$6.40\times 10^{-12}$
NIPA2	$8.06\times 10^{-5}$	$1.87\times 10^{-1}$	$4.06\times 10^{-1}$	$1.10\times 10^{-6}$
PPP5C	$5.39\times 10^{-5}$	$2.17\times 10^{-1}$	$5.82\times 10^{-1}$	$2.97\times 10^{-6}$

To further verify our findings, we point out gene PPP5C. The *P*-values of this gene for metaCCA, MGAS, MAT, and TWT are $5.39\times 10^{-5}$, 0.22, 0.58, and $2.97\times 10^{-6}$, respectively. Only TWT reaches the significance level of $3.85\times 10^{-6}$. PPP5C gene has reported having biological relationship with polyunsaturated fatty acids (Krysiak et al. 2018) and is associated with cardiovascular disease (Kompa et al. 2021). Other genes, such as CAPZA1 and NIPA2, are also reported to have relationship with blood pressure (Kato et al. 2011, Surendran et al. 2016) and type 2 diabetes (Zhao et al. 2020), which are closely related to levels of fatty acids. These facts from other studies further support the discovery of significant genes by our new method.

Here, we mainly used proximity to annotate SNPs with target genes. However, linking SNPs with their closest genes may not be the best way in some situations, since regulatory SNPs do not necessarily regulate the closest genes (Gazal et al. 2022). Thus, many other computational methods have been developed to recently to link SNPs to their target genes with different types of information (Ernst et al. 2011, Javierre et al. 2016, Fulco et al. 2019). Among them, Gazal et al. (2022) evaluated and combined multiple SNPs to genes methods and developed combined S2G strategy (cS2G) as a powerful method to link SNPs with their target gene. Here, we used the cS2G method annotated results from 1000 Genomes Project European reference panel to re-annotate our polyunsaturated fatty acids data to verify our findings.

After the data pre-processing procedure similar as mentioned above, 14 477 genes were included for further analysis. Thus, the significance level was set to $3.45\times 10^{-6}(\approx 0.05/14477)$. metaCCA, MGAS, MAT, and TWT identified 25, 11, 10, and 27 significant genes. It can be seen that our proposed TWT identified most genes coherent with the result mentioned above. Among those 27 genes, 14 genes were detected significantly by both proximity annotation and cS2G method. Include the FADS genes: FADS1, FADS2, and FADS3, which were reported to have relationship with polyunsaturated fatty acids (Schaeffer et al. 2006, Rzehak et al. 2009).

## 5 Discussion

Summary statistics have become a popular method of data sharing in GWAS since individual level data are not available due to privacy and legal restriction. Although one-to-one analysis has been successful in the past decade, there is still missing heritability of complex diseases (Manolio et al. 2009). Therefore, multidimensional extension of both traits and variants may be one of the solutions to fill the gap since it combines the effect of intrinsic genetic structure and pleiotropic effect that are missing by single trait single variant analysis. Some genetic correlation estimation methods have already been proposed to understand the relationships among complex traits and multiple variants (Bulik-Sullivan et al. 2015, Wu et al. 2022b)

To mine the information hidden in the summary data, we proposed a new multiple-to-multiple test statistic called TWT by integrating the intrinsic genetic structure, pleiotropy, and potential information combinations. Besides this test, this work also makes two other useful contributions including the derivation of multivariate normal distribution for the vectorized form of *M* summary Wald test statistics, and providing a procedure to estimate the correlation coefficient between two summary Wald test statistics while adjusting for covariates. Since summary statistics are calculated and collected under different studies, the calculation of these summary statistics may also be different from study to study. Thus, a generalized estimation method for the correlation coefficient needs to be derived. We also considered other situations of summary statistics, which are commonly encountered in GWAS data. Meta-analysis summary statistics are commonly used when multiple cohorts exist in studies (Howson et al. 2017, Nolte et al. 2017). From Supplementary Part 3 in the online supplementary material, we also give general derivation of the correlation coefficient for meta-analysis and prove that our proposed estimation method still holds in the situation of meta-analysis summary statistics.

Unlike other methods directly generalized traditional method, TWT is specially designed for multiple-to-multiple situations, which can fully use the information that is ignored by other methods. Simulations also show that our proposed method outperforms other methods when the dimensions of traits and variants are high and the signals are sparse, which are commonly encountered in GWAS data. Multidimensional analysis has recently become popular not only in the area of GWAS but also in gene expression data. The corresponding study is called gene set enrichment analysis (Subramanian et al. 2005). This indicates that our proposed method may be generalized to other areas. When *q* and *m* are both large, we point out that it might take a long time to run with existing methods including TWT. The most time-consuming step is the calculation of null distribution of ${\tilde{T}}_{\eta }$. As a truncated statistic, ${\tilde{T}}_{\eta }$ does not follow traditional distribution. We use generalized χ^2^ distribution to approximate the null distribution of ${\tilde{T}}_{\eta }$ so that we can generate *P*-values arbitrarily small that satisfy GWAS stringent significance level. However, we still need to generate random samples to estimate the parameter of generalized χ^2^ distribution. Although using smaller sample size to conduct estimation of the parameters will save much time, to ensure the accuracy of our final *P*-values, we use large sample size. Large sample generation and calculation is the main reason that our proposed methods were computationally slow under high dimension situations. Thus, it is valuable to build test statistics specially designed for large *q* and *m*, such as the sliding windows strategy. Also, inflated type I error was observed in some scenarios, such as $S_{1-3}$ and $S_{2-2}$. Here, we briefly explain two reasons for the inflation. First, as we mentioned, we use generalized χ^2^ distribution to approximate ${\tilde{T}}_{\eta }$. Although we generate large number of random samples to ensure that our estimation is accurate, errors may still occur. This will cause the inflated type I error rate in some of our scenarios. Second, we use Cauchy combination method multiple times to construct our final test statistics. Although Cauchy combination method is very convenient for combing *P*-values without complex bootstrap procedures, it may have inflated type I error rate because of the approximation error. Generally speaking, the inflation is related to the correlation structure of the *P*-values and significance level (Liu et al. 2019). Thus, inflated type I error rate occurs in some of our scenarios. However, this inflation will not be a serious problem based on the simulations in other reference (Liu and Xie 2020) and becomes smaller as the significant level becomes smaller (Liu et al. 2019). Although the main object of this article is not to identify which individual SNPs affected these analyzed phenotypes or which individual phenotypes were affected by these analyzed SNPs, by further observing the computation process of our test statistics, we can gain insights of the detailed biological process. Our final test statistics is a combination of three *P*-values $P_{1}$, $P_{2}$, and $P_{3}$. By observing which of these *P*-values are smallest, we can determine which effect is most likely to exist. For example, we observe that $P_{1}$ is the smallest among these three *P*-values, this indicates that multiple SNPs may associate with one particular phenotype. To find this phenotype, we can further decompose $P_{1}$. As $P_{1}$ is the combination of multiple *P*-values each representing one phenotype, we can further find which *P*-value is the smallest, then, we can identify which phenotype is most likely to have association with multiple SNPs. Further studies are envisioned to investigate these areas.

## Supplementary Material

btad182_Supplementary_Data
