# Supplementary material for: Summary statistics-based association test for identifying the pleiotropic effects with set of genetic variants
Source: Bioinformatics. 2023 Apr 7;39(4):btad182. doi: 10.1093/bioinformatics/btad182 (PMC10115469; doi:10.1093/bioinformatics/btad182)
Supplement: btad182_Supplementary_Data [file btad182_supplementary_data.pdf]

Supplemental Materials on "Summary statistics-based association test for identifying the pleiotropic effects with set of genetic variants"

Deliang Bu, Xiao Wang, Qizhai Li

In this Supplemental Materials, we provide the following results. Part 1 gives the derivation of asymptotic multivariate normal distribution of vec($\boldsymbol{Z}$). In Part 2, we show the technical details of estimating the correlation coefficients of summary Wald statistics. Part 3 focuses on estimating correlation coefficients for meta-analysis summary statistics. Part 4 lists detailed simulation settings of our simulations in main text. Part 5 provide curves of power under different significance levels. Part 6 lists all significance genes detected by TWT. Part 7 provides the Q-Q plots for metaCCA, MGAS, MAT and TWT with polyunsaturated fatty acids data. Part 8 provides the Venn diagram for real data analysis. Part 9 provides the R code for calculating TWT.

Part 1. Asymptotic multivariate normal distribution of vec($\boldsymbol{Z}$)

Suppose there are n subjects in a genome-wide association study. For each subject, q quantitative phenotypes, m single nucleotide polymorphisms (SNPs) and s covariates are measured. Let the observations of the jth phenotype be $\boldsymbol{y}_j = (y_{1j}, y_{2j}, \cdots, y_{nj})^\top$, and those of the kth SNP be $\boldsymbol{g}_k = (g_{1k}, g_{2k}, \cdots, g_{nk})^\top$, $j = 1, 2, \cdots, p$ and $k = 1, 2, \cdots, m$. Let $\boldsymbol{C}$ be $n \times (s+1)$ matrix representing s covariates with the first column all equaling 1 corresponding to the intercept term. The linear regression model for the jth phenotype and kth SNP is

$$\boldsymbol{y}_j = \boldsymbol{g}_k \beta_{jk} + \boldsymbol{C}\boldsymbol{\gamma}_j + \boldsymbol{\varepsilon}_j, j = 1, 2, \cdots q, k = 1, 2, \cdots, m,$$

where $\boldsymbol{\gamma}_j = (\gamma_{j0}, \gamma_{j1}, \cdots, \gamma_{js})^\top$ and β_{jk} is the regression coefficient. We assume $\boldsymbol{\mathcal{E}} = (\boldsymbol{\varepsilon}_1, \boldsymbol{\varepsilon}_2, \cdots, \boldsymbol{\varepsilon}_q) = (\varepsilon_{ij})_{n\times q}$, an $n \times q$-dimensional matrix, and $\text{vec}(\boldsymbol{\mathcal{E}}^\top) \sim N(\boldsymbol{0}_{nq}, \boldsymbol{I}_n \otimes \boldsymbol{\Delta})$. Here $\boldsymbol{I}_n$ denote the n-dimensional identity matrix, and $\boldsymbol{\Delta}$ is the covariance matrix of $(\varepsilon_{i1}, \varepsilon_{i2}, \cdots \varepsilon_{iq})^\top$. This setting is commonly used in pleiotropy analysis (Liu and Lin, 2019; Zhu et al., 2015)

Now we derive the Wald test statistic $Z_{jk}, j = 1, 2, \cdots q, k = 1, 2, \cdots m$. Since the Wald test statistic is the maximum likelihood estimate divided by its variance, we start by considering the maximum likelihood estimate, i.e. the least square estimator of β_{jk}. Let $\boldsymbol{X} = (\boldsymbol{g}_k, \boldsymbol{C})$. The maximum likelihood estimate of $(\beta_{jk}, \boldsymbol{\gamma}_j^\top)^\top$ is $(\hat{\beta}_{jk}, \hat{\boldsymbol{\gamma}}_j^\top)^\top = (\boldsymbol{X}^\top\boldsymbol{X})^{-1}\boldsymbol{X}^\top\boldsymbol{y}_j$. According to the facts that

$$\boldsymbol{X}^\top\boldsymbol{X} = \begin{pmatrix} \boldsymbol{g}_k^\top\boldsymbol{g}_k & \boldsymbol{g}_k^\top\boldsymbol{C} \\ \boldsymbol{C}^\top\boldsymbol{g}_k & \boldsymbol{C}^\top\boldsymbol{C} \end{pmatrix}$$

and the first row of $(\boldsymbol{X}^\top\boldsymbol{X})^{-1}$ is

$$\Big([\boldsymbol{g}_k^\top\boldsymbol{g}_k-\boldsymbol{g}_k^\top\boldsymbol{C}(\boldsymbol{C}^\top\boldsymbol{C})^{-1}\boldsymbol{C}^\top\boldsymbol{g}_k]^{-1},-[\boldsymbol{g}_k^\top\boldsymbol{g}_k-\boldsymbol{g}_k^\top\boldsymbol{C}(\boldsymbol{C}^\top\boldsymbol{C})^{-1}\boldsymbol{C}^\top\boldsymbol{g}_k]^{-1}\boldsymbol{g}_k^\top\boldsymbol{C}(\boldsymbol{C}^\top\boldsymbol{C})^{-1}\Big),$$

the maximum likelihood estimate of β_{jk} is

$$\hat{\beta}_{jk}=[\boldsymbol{g}_k^\top\boldsymbol{g}_k-\boldsymbol{g}_k^\top\boldsymbol{C}(\boldsymbol{C}^\top\boldsymbol{C})^{-1}\boldsymbol{C}^\top\boldsymbol{g}_k]^{-1}\boldsymbol{g}_k^\top[\boldsymbol{I}_n-\boldsymbol{C}(\boldsymbol{C}^\top\boldsymbol{C})^{-1}\boldsymbol{C}^\top]\boldsymbol{y}_j.$$

Denote $\boldsymbol{H}=\boldsymbol{C}(\boldsymbol{C}^\top\boldsymbol{C})^{-1}\boldsymbol{C}^\top$. Then we have

$$\hat{\beta}_{jk}=[\boldsymbol{g}_k^\top(\boldsymbol{I}_n-\boldsymbol{H})\boldsymbol{g}_k]^{-1}\boldsymbol{g}_k^\top(\boldsymbol{I}_n-\boldsymbol{H})\boldsymbol{y}_j.$$

Plugging in the value of $\boldsymbol{y}_j$, we can get that

$$\hat{\beta}_{jk}=[\boldsymbol{g}_k^\top(\boldsymbol{I}_n-\boldsymbol{H})\boldsymbol{g}_k]^{-1}\boldsymbol{g}_k^\top(\boldsymbol{I}_n-\boldsymbol{H})(\boldsymbol{g}_k\beta_{jk}+\boldsymbol{C}\boldsymbol{\gamma}_j+\boldsymbol{\varepsilon}_j).$$

Because $(\boldsymbol{I}_n-\boldsymbol{H})\boldsymbol{C}=\boldsymbol{0}_{n\times(s+1)}$, a $n\times(s+1)$-dimensional matrix with all the elements being 0,

$$\hat{\beta}_{jk}=[\boldsymbol{g}_k^\top(\boldsymbol{I}_n-\boldsymbol{H})^\top(\boldsymbol{I}_n-\boldsymbol{H})\boldsymbol{g}_k]^{-1}\boldsymbol{g}_k^\top(\boldsymbol{I}_n-\boldsymbol{H})(\boldsymbol{g}_k\beta_{jk}+\boldsymbol{\varepsilon}_j).$$

Let $\boldsymbol{\beta}=(\beta_{11},\beta_{12},\cdots,\beta_{qm})$, $\boldsymbol{A}_k=[\boldsymbol{g}_k^\top(\boldsymbol{I}_n-\boldsymbol{H})^\top(\boldsymbol{I}_n-\boldsymbol{H})\boldsymbol{g}_k]^{-1}\boldsymbol{g}_k^\top(\boldsymbol{I}_n-\boldsymbol{H}), k=1,2,\cdots m,$

$$\boldsymbol{A}=\begin{pmatrix}\boldsymbol{A}_1\\ \boldsymbol{A}_2\\ \vdots\\ \boldsymbol{A}_m\end{pmatrix},\quad \boldsymbol{A}_q=\begin{pmatrix}\boldsymbol{A}&&&\\&\boldsymbol{A}&&\\&&\ddots&\\&&&\boldsymbol{A}\end{pmatrix},$$

where $\boldsymbol{A}_q$ is a block diagnoal matrix with q matrices of $\boldsymbol{A}$ being the diagnoal elements. The maximum likelihood estimate of $\boldsymbol{\beta}$, denote as $\hat{\boldsymbol{\beta}}$, can be expressed as the linear combination of $\text{vec}(\mathcal{E})$, that is, $\hat{\boldsymbol{\beta}}=\boldsymbol{\beta}+\boldsymbol{A}_q\text{vec}(\mathcal{E})$, Since $\boldsymbol{\beta}$ is a vector of constant and $\mathcal{E}$ jointly follows multivariate normal distribution, $\hat{\boldsymbol{\beta}}$ follows the multivariate normal distribution.

The Wald test statistic Z_{jk} can be written as

$$Z_{jk}=\frac{\hat{\beta}_{jk}}{\hat{\sigma}^2(\hat{\beta}_{jk})},$$

where $\hat{\sigma}^2(\hat{\beta}_{jk})$ is a consistent estimate of the variance of $\hat{\beta}_{jk}$ which converges to a constant in probability. By Slustky theorem, we can conclude that $\text{vec}(\boldsymbol{Z})$ jointly follows a multivariate normal distribution under the null hypothesis.

Part 2. Estimating the correlation coefficients of summary Wald statistics in linear regression model with covariates

We derive the pairwise correlation coefficient between two Wald test statistics Z_{jk} and $Z_{j'k'}$ under the linear regression model with covariates, where $j, j' = 1, 2, \cdots, q$ and $k, k' = 1, 2, \cdots, m$. We now derive the expression of Z_{jk} and $Z_{j'k'}$. Recall that in Part 1, we have

$$\hat{\beta}_{jk} = [\boldsymbol{g}_k^\top (\boldsymbol{I}_n - \boldsymbol{H}) \boldsymbol{g}_k]^{-1} \boldsymbol{g}_k^\top (\boldsymbol{I}_n - \boldsymbol{H}) \boldsymbol{y}_j.$$

Since $(\boldsymbol{I}_n - \boldsymbol{H})^2 = \boldsymbol{I}_n - \boldsymbol{H}, (\boldsymbol{I}_n - \boldsymbol{H})^\top = \boldsymbol{I}_n - \boldsymbol{H}$, we can rewrite it as

$$\hat{\beta}_{jk} = [\boldsymbol{g}_k^\top (\boldsymbol{I}_n - \boldsymbol{H})^\top (\boldsymbol{I}_n - \boldsymbol{H}) \boldsymbol{g}_k]^{-1} \boldsymbol{g}_k^\top (\boldsymbol{I}_n - \boldsymbol{H})^\top \boldsymbol{y}_j.$$

It can be seen that $\boldsymbol{h}_k = (\boldsymbol{I}_n - \boldsymbol{H}) \boldsymbol{g}_k$ is the residuals after regressing the genetic variable $\boldsymbol{g}_k$ on covariates $\boldsymbol{C}$, then

$$\hat{\beta}_{jk} = (\boldsymbol{h}_k^\top \boldsymbol{h}_k)^{-1} \boldsymbol{h}_k^\top \boldsymbol{y}_j.$$

Thus the Wald test statistics is obtained via $\hat{\beta}_{jk}$ dividing by its estimated variance as

$$Z_{jk} = \frac{\hat{\beta}_{jk}}{s^2(\hat{\beta}_{jk})} = \boldsymbol{h}_k^\top \boldsymbol{y}_j / \sqrt{\hat{\sigma}_j^2 \boldsymbol{h}_k^\top \boldsymbol{h}_k},$$

where $\hat{\sigma}_j^2$ is the consistent estimate of σ_j, the unknown variance of the random error. For convenience, we replace Z_{jk} with the true variance σ_j^2 and rewrite it as

$$Z_{jk} = \frac{\hat{\beta}_{jk}}{s^2(\hat{\beta}_{jk})} = \boldsymbol{h}_k^\top \boldsymbol{y}_j / \sqrt{\sigma_j^2 \boldsymbol{h}_k^\top \boldsymbol{h}_k}.$$

Similarly, we have $Z_{j'k'}$

$$Z_{j'k'} = \boldsymbol{h}_{k'}^\top \boldsymbol{y}_{j'} / \sqrt{\sigma_{j'}^2 \boldsymbol{h}_{k'}^\top \boldsymbol{h}_{k'}}.$$

Finally, we get the correlation coefficient of Z_{jk} and $Z_{j'k'}$ as

$$\text{corr}(Z_{jk}, Z_{j'k'}) = \rho_{jj'} \times \frac{\boldsymbol{h}_k^\top \boldsymbol{h}_{k'}}{\sqrt{\boldsymbol{h}_k^\top \boldsymbol{h}_k} \sqrt{\boldsymbol{h}_{k'}^\top \boldsymbol{h}_{k'}}}.$$

Since $\boldsymbol{h}_k$ and $\boldsymbol{h}_{k'}$ is the residual for regressing $\boldsymbol{g}_k$ and $\boldsymbol{g}_{k'}$ on covariates $\boldsymbol{C}$ which have zero means, it can be concluded that

$$\text{corr}(Z_{jk}, Z_{j'k'}) = \rho_{jj'} \theta_{kk'},$$

Here $\theta_{kk'}$ is the partial correlation coefficient between genetic variable $\boldsymbol{g}_k$ and $\boldsymbol{g}_{k'}$ given covariates $\boldsymbol{C}$.

Part 3. Estimating the correlation coefficients of meta-analysis summary statistics

The meta-analysis summary statistics are commonly used when multiple cohorts data are available. Suppose we perform univariate GWAS analysis of 2 phenotypes in H cohorts with sample size $n_1, n_2, \cdots n_H$. Assume that the between-phenotype correlation coefficient is the same across the cohorts, denote it by $\rho_{jj'}$. Let $Z_{jk}^{(h)}$ be the Wald test statistics of the hth cohort for the jth phenotype and kth SNPs, and $Z_{j'k'}^{(h)}$ be the Wald test statistics of the j'th phenotype and the k'th SNP, wheres $h = 1, 2, \cdots H, j, j' = 1, 2, \cdots, q$ and $k, k' = 1, 2, \cdots, m$. The meta-analysis z-scores can be calculated as

$$Z_{jk} = w_1 Z_{jk}^{(1)} + w_2 Z_{jk}^{(2)} + \cdots w_H Z_{jk}^{(H)}, Z_{j'k'} = w_1 Z_{j'k'}^{(1)} + w_2 Z_{j'k'}^{(2)} + \cdots w_H Z_{j'k'}^{(H)},$$

where $w_1, w_2, \cdots w_H$ are weights calculated based on sample size (Willer et al., 2010) and satisfy $w_1^2 + w_2^2 + \cdots w_H^2 = 1$. We can calculate the correlation coefficient of Z_{jk} and $Z_{j'k'}$ as

$$\text{corr}(Z_{jk}, Z_{j'k'}) = w_1^2 \rho_{jj'} \theta_{kk'}^{(1)} + w_2^2 \rho_{jj'} \theta_{kk'}^{(2)} + \cdots w_H^2 \rho_{jj'} \theta_{kk'}^{(H)}.$$

Here $\theta_{kk'}^{(h)}, h = 1, 2, \cdots H$ is the partial correlation coefficient of genetic variable given covariates in cohort h. Although $\theta_{kk'}^{(h)}, i = 1, 2, \cdots H$ are different from each other from cohort to cohort, they are all determined by the genetic structure of the target population. Thus it is reasonable to assume that they converge to the same value $\theta_{kk'}$ as the sample size of each cohort $n_1, n_2, \cdots n_H \to \infty$. Thus we can conclude that $\text{corr}(Z_{jk}, Z_{j'k'}) = \rho_{jj'} \theta_{kk'}$. Thus the estimation process can be done as mentioned in the main text.

Part 4. Simulation Settings

Table 1: Detailed scenario configurations of S_{1-1} to S_{2-3}. Gene represents the gene used as genotype in the regression model, q stands for the dimension of phenotype, and $\boldsymbol{\Delta}_\rho$ is the corresponding phenotype correlation matrix. Pattern of β_{jk} stands for the detailed configuration of regression coefficients.

	Gene	q	$\boldsymbol{\Delta}_\rho$	Pattern of β_{jk}
S_{1-1}	FADS2	6	Real data correlation matrix	NONE
S_{1-2}	GNB1	6	Real data correlation matrix	NONE
S_{1-3}	SGIP1	6	Real data correlation matrix	NONE
S_{2-1}	ACOT7	10	Autoregressive correlation matrix $\rho = 0.1$	NONE
S_{2-2}	ACOT7	10	Autoregressive correlation matrix $\rho = 0.5$	NONE
S_{2-3}	ACOT7	10	Autoregressive correlation matrix $\rho = 0.8$	NONE

Table 2: Detailed scenario configurations of S_{1-1-1} to S_{1-3-3}. Gene represents the gene used as genotype in the regression model, q stands for the dimension of phenotype, and $\boldsymbol{\Delta}_\rho$ is the corresponding phenotype correlation matrix. The pattern of β_{jk} stands for the detailed configuration of regression coefficients.

	Gene	q	$\boldsymbol{\Delta}_\rho$	Pattern of β_{jk}
S_{1-1-1}	FADS2	6	Real data correlation matrix	SINGLE
S_{1-1-2}	FADS2	6	Real data correlation matrix	COLUMN
S_{1-1-3}	FADS2	6	Real data correlation matrix	ROW
S_{1-2-1}	GNB1	6	Real data correlation matrix	SINGLE
S_{1-2-2}	GNB1	6	Real data correlation matrix	COLUMN
S_{1-2-3}	GNB1	6	Real data correlation matrix	ROW
S_{1-3-1}	SGIP1	6	Real data correlation matrix	SINGLE
S_{1-3-2}	SGIP1	6	Real data correlation matrix	COLUMN
S_{1-3-3}	SGIP1	6	Real data correlation matrix	ROW

Table 3: Detailed scenario configurations of S_{2-1-1} to S_{2-3-3}. Gene represents the gene used as genotype in the regression model, q stands for the dimension of phenotype, and $\mathbf{\Delta}_\rho$ is the corresponding phenotype correlation matrix. The pattern of β_{jk} stands for the detailed configuration of regression coefficients.

	Gene	q	$\mathbf{\Delta}_\rho$	Pattern of β_{jk}
S_{2-1-1}	ACOT7	10	Auto-regressive correlation matrix $\rho = 0.1$	SINGLE
S_{2-1-2}	ACOT7	10	Auto-regressive correlation matrix $\rho = 0.1$	COLUMN
S_{2-1-3}	ACOT7	10	Auto-regressive correlation matrix $\rho = 0.1$	ROW
S_{2-2-1}	ACOT7	10	Auto-regressive correlation matrix $\rho = 0.5$	SINGLE
S_{2-2-2}	ACOT7	10	Auto-regressive correlation matrix $\rho = 0.5$	COLUMN
S_{2-2-3}	ACOT7	10	Auto-regressive correlation matrix $\rho = 0.5$	ROW
S_{2-3-1}	ACOT7	10	Auto-regressive correlation matrix $\rho = 0.8$	SINGLE
S_{2-3-2}	ACOT7	10	Auto-regressive correlation matrix $\rho = 0.8$	COLUMN
S_{2-3-3}	ACOT7	10	Auto-regressive correlation matrix $\rho = 0.8$	ROW

Part 5. Curves of power under different significance levels

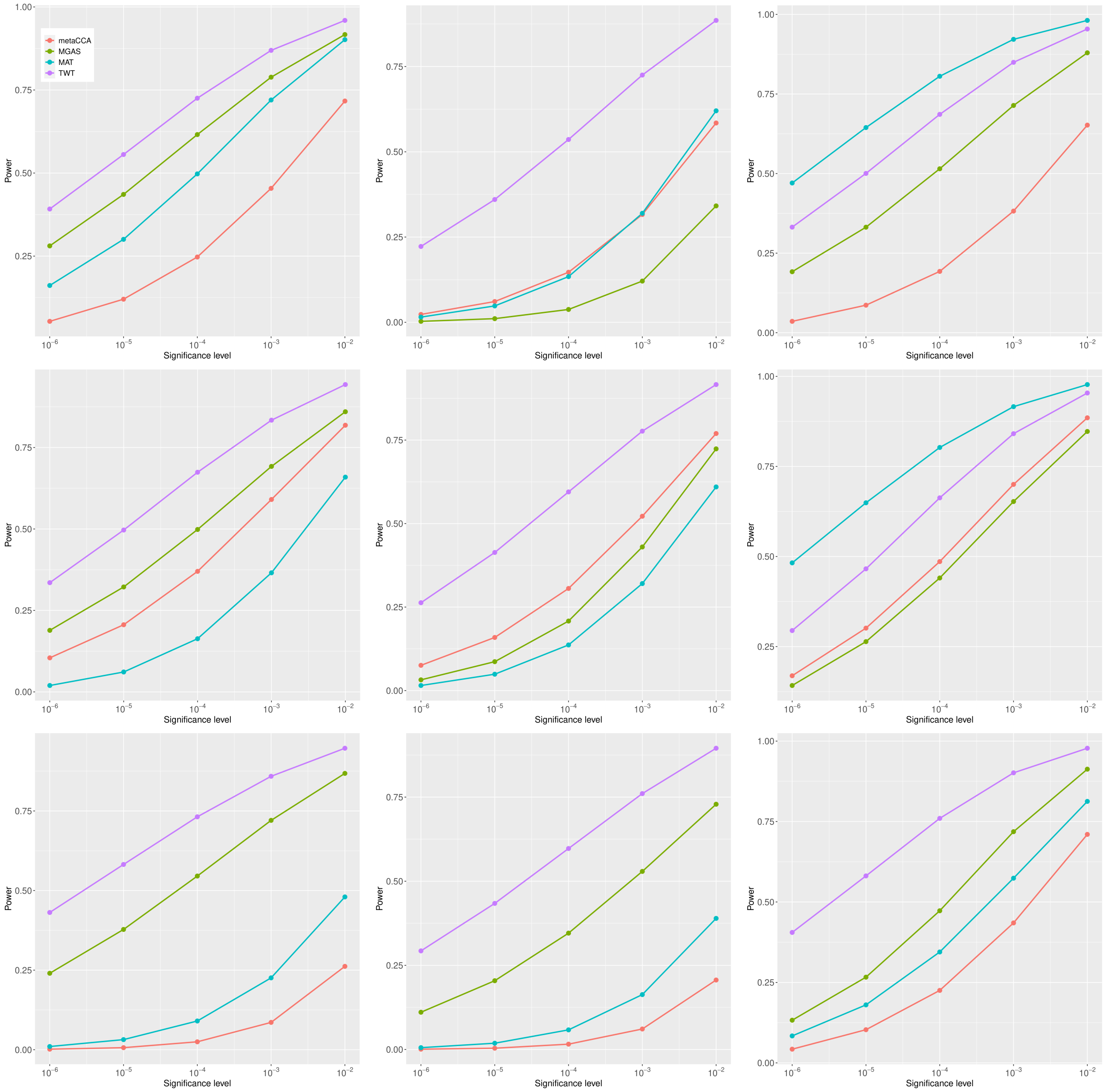

Figure 1: Power curves of metaCCA(red), MGAS (green), MAT (blue), and TWT (purple) with different significance levels under scenarios S_{1-1-1} to S_{1-3-3}. The x-axis is significance level with 10^{-6}, 10^{-5}, 10^{-4}, 10^{-3}, 10^{-2} and y-axis is the empirical power.

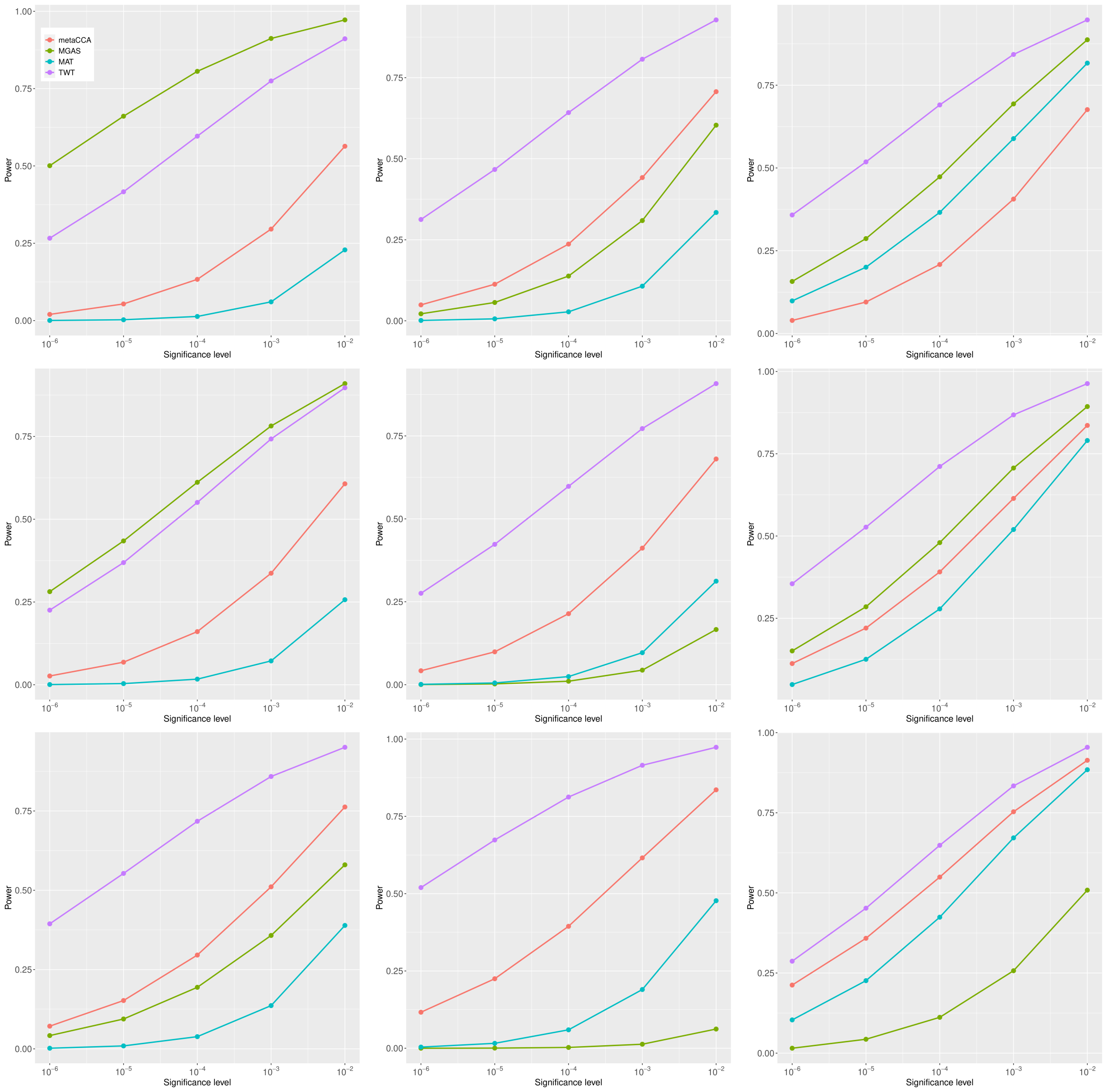

Figure 2: Power curves of metaCCA(red), MGAS (green), MAT (blue), and TWT (purple) with different significance levels under scenarios S_{2-1-1} to S_{2-3-3}. The x-axis is significance level with 10^{-6}, 10^{-5}, 10^{-4}, 10^{-3}, 10^{-2} and y-axis is the empirical power.

Part 6. Significant genes detected by TWT

Table 4: P-values of genes that are detected significant by TWT, where the genes only detected significant by TWT are marked in boldface.

Gene	metaCCA	MGAS	MAT	TWT
LINC01225	5.09×10^{-65}	5.27×10^{-1}	2.77×10^{-1}	$< 10^{-14}$
**LINC01226**	5.13×10^{-5}	9.00×10^{-1}	3.08×10^{-1}	6.60×10^{-7}
NRD1	1.54×10^{-15}	6.67×10^{-1}	4.35×10^{-1}	3.32×10^{-13}
**GNG12**	4.13×10^{-4}	2.88×10^{-1}	8.82×10^{-1}	6.47×10^{-10}
**CAPZA1**	3.08×10^{-4}	2.82×10^{-1}	6.45×10^{-1}	1.96×10^{-6}
ATP1A4	1.77×10^{-7}	1.03×10^{-1}	8.35×10^{-1}	9.03×10^{-10}
LAMC2	3.58×10^{-24}	9.48×10^{-1}	8.13×10^{-1}	$< 10^{-14}$
LOC284950	6.11×10^{-34}	7.91×10^{-1}	5.33×10^{-1}	$< 10^{-14}$
CCNT2-AS1	2.97×10^{-10}	3.27×10^{-2}	2.99×10^{-3}	1.24×10^{-10}
KCNH7	1.83×10^{-20}	9.00×10^{-1}	3.66×10^{-1}	$< 10^{-14}$
USP37	4.13×10^{-17}	8.84×10^{-1}	7.08×10^{-1}	2.14×10^{-14}
NUP210	3.46×10^{-11}	1.08×10^{-2}	7.33×10^{-4}	4.33×10^{-10}
TRPC1	3.26×10^{-26}	5.24×10^{-1}	5.50×10^{-1}	$< 10^{-14}$
COMMD2	5.73×10^{-13}	2.17×10^{-1}	4.28×10^{-1}	9.38×10^{-11}
CCDC50	1.93×10^{-48}	1.63×10^{-1}	2.45×10^{-1}	$< 10^{-14}$
**SRP72**	1.91×10^{-5}	4.14×10^{-2}	3.09×10^{-2}	9.25×10^{-8}
KLHL8	5.69×10^{-37}	9.45×10^{-1}	7.98×10^{-1}	$< 10^{-14}$
SNCA	1.64×10^{-11}	9.85×10^{-1}	8.76×10^{-1}	1.34×10^{-9}
LOC101927087	3.03×10^{-23}	2.54×10^{-1}	7.25×10^{-2}	$< 10^{-14}$
LYSMD3	2.05×10^{-25}	1.33×10^{-1}	1.21×10^{-1}	$< 10^{-14}$
**AQPEP**	3.93×10^{-6}	6.34×10^{-1}	3.50×10^{-1}	3.61×10^{-9}
MARCH3	3.23×10^{-8}	3.80×10^{-1}	5.15×10^{-1}	2.24×10^{-6}
**CPEB4**	8.56×10^{-6}	2.78×10^{-1}	1.71×10^{-1}	1.24×10^{-6}
ELOVL2	3.17×10^{-3}	4.05×10^{-5}	3.14×10^{-12}	1.97×10^{-8}
ELOVL2-AS1	1.48×10^{-7}	1.94×10^{-3}	7.58×10^{-4}	2.63×10^{-9}
ZNRD1-AS1	1.11×10^{-24}	3.34×10^{-1}	5.39×10^{-3}	$< 10^{-14}$
ZNRD1	3.71×10^{-29}	4.24×10^{-1}	4.14×10^{-2}	$< 10^{-14}$

PLG	1.45×10^{-22}	3.05×10^{-1}	4.28×10^{-1}	$< 10^{-14}$
MMD2	1.90×10^{-6}	6.81×10^{-1}	9.64×10^{-1}	5.95×10^{-7}
INMT-FAM188B	2.89×10^{-6}	1.27×10^{-1}	6.30×10^{-2}	9.17×10^{-7}
**EGFR**	3.68×10^{-5}	7.92×10^{-1}	6.33×10^{-1}	3.17×10^{-11}
IFRD1	1.99×10^{-7}	1.01×10^{-1}	5.15×10^{-1}	2.78×10^{-8}
**KAT6A**	1.45×10^{-3}	4.42×10^{-1}	2.65×10^{-1}	5.41×10^{-7}
LOC101929528	5.13×10^{-17}	9.30×10^{-1}	8.73×10^{-1}	$< 10^{-14}$
**DPYS**	1.85×10^{-3}	7.82×10^{-3}	5.43×10^{-3}	1.32×10^{-6}
TNFRSF11B	2.44×10^{-7}	5.90×10^{-1}	5.30×10^{-1}	2.60×10^{-6}
IFT74	5.75×10^{-18}	9.73×10^{-1}	6.08×10^{-1}	$5.56 \times < 10^{-14}$
DAPK1	6.77×10^{-12}	2.82×10^{-1}	2.06×10^{-1}	$< 10^{-14}$
SPTLC1	1.21×10^{-20}	1.30×10^{-1}	5.61×10^{-1}	$< 10^{-14}$
CWF19L1	3.45×10^{-8}	7.65×10^{-1}	5.57×10^{-1}	1.70×10^{-7}
BLOC1S2	7.01×10^{-8}	3.48×10^{-1}	9.14×10^{-2}	1.09×10^{-6}
MICAL2	2.15×10^{-13}	6.94×10^{-1}	5.96×10^{-2}	6.32×10^{-13}
PHF21A	2.60×10^{-14}	5.64×10^{-1}	5.31×10^{-1}	3.00×10^{-11}
MYRF	4.25×10^{-90}	6.32×10^{-45}	3.41×10^{-43}	$< 10^{-14}$
TMEM258	1.01×10^{-65}	3.51×10^{-45}	1.57×10^{-48}	$< 10^{-14}$
FADS1	2.11×10^{-66}	5.31×10^{-45}	2.17×10^{-56}	$< 10^{-14}$
FADS2	4.98×10^{-69}	3.28×10^{-43}	7.84×10^{-54}	$< 10^{-14}$
FADS3	5.59×10^{-21}	4.98×10^{-16}	3.49×10^{-21}	$< 10^{-14}$
PAAF1	8.41×10^{-21}	7.85×10^{-1}	4.80×10^{-1}	$< 10^{-14}$
POLD3	1.33×10^{-9}	7.16×10^{-1}	8.97×10^{-1}	7.80×10^{-10}
GRM5	9.47×10^{-21}	4.36×10^{-1}	1.99×10^{-1}	$< 10^{-14}$
TMEM117	3.42×10^{-14}	6.37×10^{-1}	1.25×10^{-1}	1.21×10^{-9}
ARHGEF25	3.27×10^{-4}	4.66×10^{-7}	1.34×10^{-5}	1.59×10^{-6}
IRAK3	9.99×10^{-9}	6.24×10^{-1}	2.12×10^{-1}	1.42×10^{-7}
FZD10-AS1	5.37×10^{-11}	4.33×10^{-2}	3.43×10^{-1}	1.06×10^{-11}
PHF11	2.08×10^{-6}	9.54×10^{-1}	7.50×10^{-1}	8.92×10^{-8}
RALGAPA1	5.37×10^{-16}	5.35×10^{-2}	1.29×10^{-3}	$< 10^{-14}$
**EML5**	3.37×10^{-5}	9.03×10^{-2}	7.83×10^{-4}	6.40×10^{-12}
LINC00521	1.29×10^{-7}	3.71×10^{-1}	7.89×10^{-1}	6.79×10^{-10}
**NIPA2**	8.06×10^{-5}	1.87×10^{-1}	4.06×10^{-1}	1.10×10^{-6}
VPS13C	8.48×10^{-7}	7.91×10^{-1}	5.23×10^{-1}	2.27×10^{-13}

CCP110	2.16×10^{-12}	6.22×10^{-1}	1.56×10^{-1}	7.23×10^{-12}
AP2B1	5.73×10^{-8}	7.43×10^{-1}	7.67×10^{-1}	1.21×10^{-10}
CCDC57	5.59×10^{-33}	9.96×10^{-1}	8.60×10^{-1}	$< 10 - 14$
SMCHD1	7.18×10^{-8}	1.92×10^{-1}	6.32×10^{-2}	6.87×10^{-7}
PIK3C3	1.07×10^{-8}	7.94×10^{-1}	6.82×10^{-1}	9.51×10^{-7}
DSEL	5.05×10^{-49}	3.69×10^{-1}	5.98×10^{-1}	$< 10^{-14}$
MAP1S	7.46×10^{-12}	5.86×10^{-1}	4.63×10^{-1}	1.07×10^{-9}
ZNF229	2.38×10^{-48}	6.26×10^{-1}	4.52×10^{-1}	$< 10^{-14}$
**PPP5C**	5.39×10^{-5}	2.17×10^{-1}	5.82×10^{-1}	2.97×10^{-6}
GPCPD1	1.29×10^{-73}	6.99×10^{-1}	2.79×10^{-1}	$< 10^{-14}$
KRTAP26-1	2.22×10^{-50}	6.40×10^{-1}	8.26×10^{-1}	$< 10^{-14}$
UBE2G2	1.14×10^{-9}	3.87×10^{-1}	8.92×10^{-1}	3.05×10^{-9}

Part 7. Q-Q plots for metaCCA, MGAS, MAT, and TWT

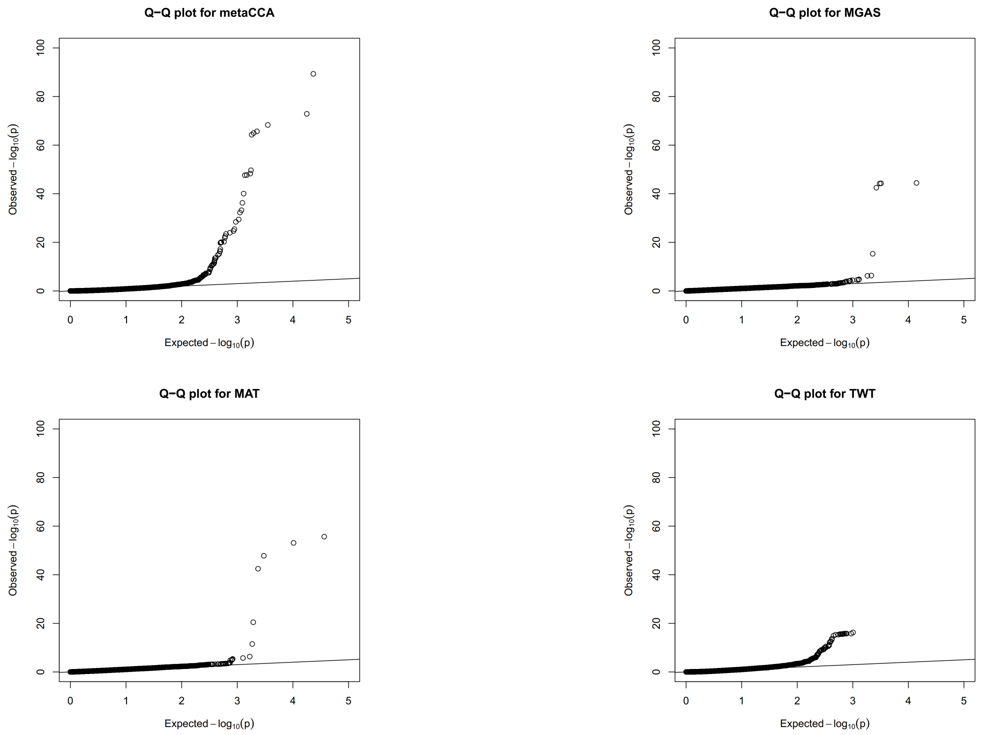

Figure 3: Q-Q plots for metaCCA, MGAS, MAT, and TWT tests p-values for the polyunsaturated fatty acids data. Note that due to the numerical precision limits of the built in function in R, TWT cannot generate p-values smaller than 10^{-14}.

Part 8. Venn diagram of significant genes detected by metaCCA, MGAS, MAT, and TWT

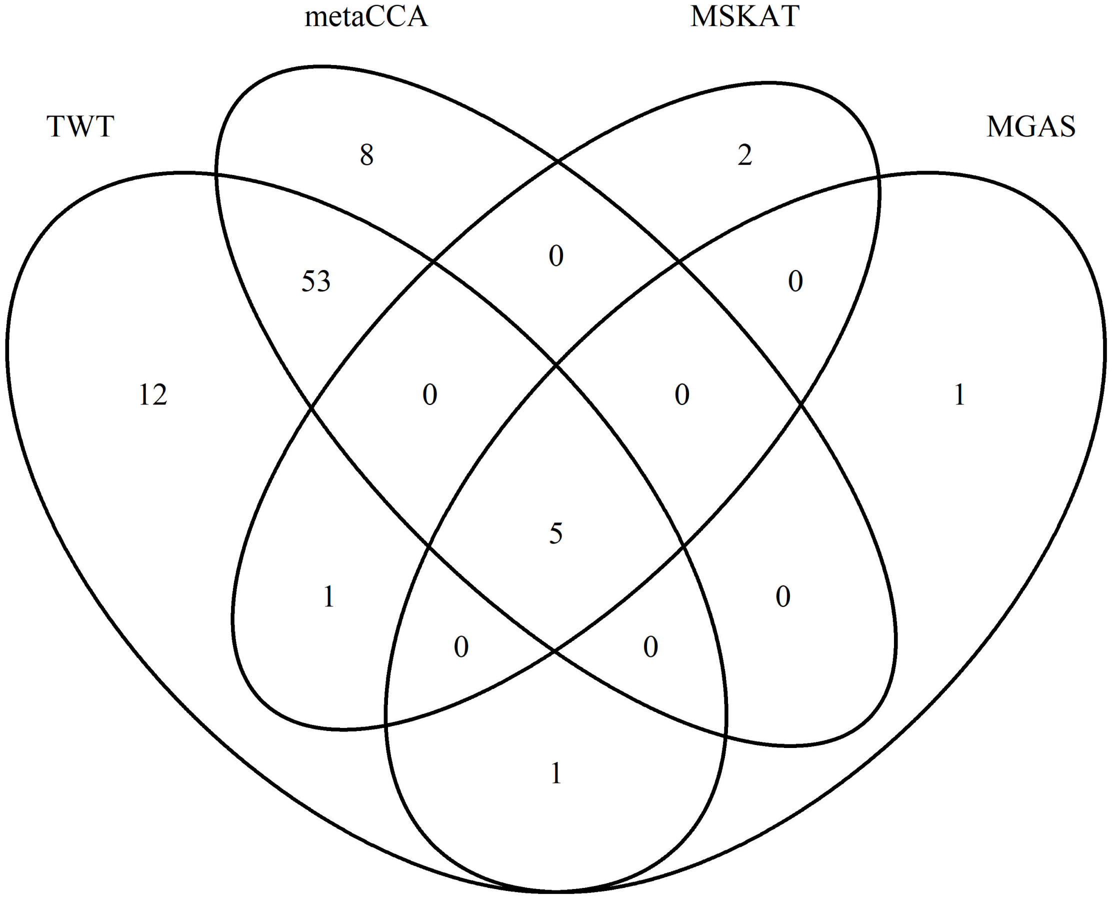

Figure 4: The Venn diagram of significant genes detected by TWT, metaCCA, MAT and MGAS.

Part 9. R code for TWT

```
#' TWT
#'
#' This is the method of TWT.
#' @param z_mat is the matrix of z_scores with row number of rows stands
   for number of phenotypes and number of columns stands for the number
   of variants.
#' @param est_genetic_cor Estimated correlation matrix of genetic
   variants.
#' @param est_pheno_cor Estimated correlation matrix of phenotypes.
#' @param cutoff_value Set Omega.
#' @param coefficient_matrix Calculated based on function approximate_
   distribution_coefficient_estimate_T3.
#' @returns p_value_final P_value of TWT.
#' @returns p_1 P_value of T_1.
#' @returns p_2 P_value of T_2.
#' @returns p_3 P_value of T_3.
#' @export
#' @examples
#' z_mat<-MASS::mvrnorm(5,mu=rep(0,5), Sigma = diag(nrow = 5, ncol = 5))
#' null_distribution<-ThreeWayTest::generate_null_distribution_T3(m=25,
#' n=1000,cov_mat=diag(nrow = 25,ncol= 25), cutoff_value=c
   (0.2,0.4,0.6,0.8,1))
#' coefficient_matrix<-ThreeWayTest::approximate_distribution_coefficient
   _estimate_T3(null_distribution)
#' ThreeWayTest::TWT(z_mat=z_mat, est_genetic_cor=diag(nrow = 5, ncol =
   5),
#' est_pheno_cor=diag(nrow = 5, ncol = 5), cutoff_value=c
   (0.2,0.4,0.6,0.8,1),
#' coefficient_matrix=coefficient_matrix)

TWT<-function(z_mat,
              est_genetic_cor,
              est_pheno_cor,
              cutoff_value,
              coefficient_matrix){
```

```
  number_of_snp<-ncol(z_mat)
  number_of_pheno<-nrow(z_mat)
  pvalue_genetic<-apply(z_mat,2,chisq_test, cov_mat=est_pheno_cor)
  cauchy_statistic_1<-(1/number_of_snp)*sum(tan((0.5-pvalue_genetic)*pi))
  p_pleiotropic<-0.5-(atan(cauchy_statistic_1)/pi)
  pvalue_pheno<-apply(z_mat,1,chisq_test, cov_mat=est_genetic_cor)
  cauchy_statistic_2<-(1/number_of_pheno)*sum(tan((0.5-pvalue_pheno)*pi))
  p_genetic_structure<-0.5-(atan(cauchy_statistic_2)/pi)
  z_vec<-as.vector(z_mat)
  est_total_cov_mat<-methods::kronecker(est_genetic_cor,est_pheno_cor)
  p_3<-T_3(z_vec,est_total_cov_mat,cutoff_value,coefficient_matrix)
  final_p_vec<-c(p_genetic_structure,p_pleiotropic,p_3)
  cauchy_statistic_final<-(1/3)*sum(tan((0.5-final_p_vec)*pi))
  p_value_final<-0.5-(atan(cauchy_statistic_final)/pi)
  return(list(p_value_final=p_value_final,
              p_1=p_genetic_structure,
              p_2=p_pleiotropic, p_3=p_3))
}
```

References

Liu, Z., and Lin, X. (2019). A geometric perspective on the power of principal component association tests in multiple phenotype studies. Journal of the American Statistical Association, 114(527), 975-990.

Zhu, X., Feng, T., Tayo, B.O., Liang, J., Young, J.H., Franceschini, N., Smith, J.A., Yanek, L.R., Sun, Y.V., Edwards, T.L., et al. (2015). Meta-analysis of correlated traits via summary statistics from GWASs with an application in hypertension. The American Journal of Human Genetics, 96(1), 21-36.
